# Characteristics of auto-quantified tumor-infiltrating lymphocytes and the prognostic value in adenocarcinoma of the esophagogastric junction, gastric adenocarcinoma, and esophageal squamous cell carcinoma

**DOI:** 10.18632/aging.205999

**Published:** 2024-07-05

**Authors:** Hao Dong, Longqing Yao, Jiahui Fan, Peipei Gao, Xiaorong Yang, Ziyu Yuan, Tiejun Zhang, Ming Lu, Xingdong Chen, Chen Suo

**Affiliations:** 1 Department of Epidemiology and Ministry of Education Key Laboratory of Public Health Safety, School of Public Health, Fudan University, Shanghai, China; 2 State Key Laboratory of Genetic Engineering, Human Phenome Institute, School of Life Sciences, Fudan University, Shanghai, China; 3 Department of Clinical Laboratory, Shanghai Fourth People’s Hospital Affiliated to Tongji University School of Medicine, Shanghai, China; 4 Fudan University Taizhou Institute of Health Sciences, Taizhou, China; 5 Clinical Epidemiology Unit, Qilu Hospital of Shandong University, Jinan, China; 6 Yiwu Research Institute of Fudan University, Yiwu, Zhejiang, China; 7 Shanghai Institute of Infectious Disease and Biosecurity, Fudan University, Shanghai, China

**Keywords:** adenocarcinoma of the esophagogastric junction, gastric adenocarcinoma, esophageal squamous cell carcinoma, tumor-infiltrating lymphocytes, survival

## Abstract

Background: Adenocarcinoma of the esophagogastric junction (AEGJ) with a specific pathological profile and poor prognosis has limited therapeutic options. Previous studies have found that TILs exhibit distinct characteristics in different tumors and are correlated with tumor prognosis. We established cellular training sets to obtain auto-quantified TILs in pathological images. And we compared the characteristics of TILs in AEGJ with those in esophageal squamous cell carcinoma (ESCC) and gastric adenocarcinoma (GAC) to gain insight into the unique immune environments of these three tumors and investigate the prognostic value of TILs in these three tumors.

Methods: Utilizing a case-control study design, we analyzed 214 AEGJ, 256 GAC, and 752 ESCC cases. The TCGA dataset was used to validate prognostic value of auto-quantified TILs. The specific cellular training sets were established by experienced pathologists to determine TILs counts. Kruskal-Wallis test and multi-variable linear regression were conducted to explore TILs characteristics. Survival analyses were performed with Kaplan-Meier method and Cox proportional hazards model.

Results: The three cellular training sets of these cancers achieved a classification accuracy of 87.55 at least. The median auto-quantified TILs of AEGJ, GAC, and ESCC cases were 4.82%, 1.92%, and 0.12%, respectively. The TILs demonstrated varied characteristics under distinctive clinicopathological traits. The higher TILs proportion was associated with better prognosis in esophagogastric cancers (all *P* < 0.05) and was an independent prognostic biomarker on AEGJ in both datasets (Taixing: HR = 0.965, 95% CI = 0.938–0.994; TCGA: HR = 0.811, 95% CI = 0.712–0.925).

Conclusions: We found variations in TILs across ESCC, GAC, and AEGJ, as assessed by image processing algorithms. Additionally, TILs in these three cancers are an independent prognostic factor. This enhances our understanding of the unique immune characteristics of the three tumors, improving patient outcomes.

## INTRODUCTION

Globally, gastric and esophageal cancers were ranked as the fourth and sixth leading causes of cancer-related deaths, and were responsible for 769,000 and 544,000 deaths in 2020, respectively [1]. Given the advanced or metastatic nature of many gastroesophageal cases at diagnosis, the overall 5-year survival rate remains less than 20% in developing countries [2, 3]. The main factors affecting gastroesophageal cancers prognosis include tumor staging and grading, treatment method, living condition, and genetic marker [4, 5]. However, as a transitional region tumor from esophageal squamous epithelium to the gastric adenoid epithelium, it is possible that the oncological principles for esophageal and gastric cancer are not directly applicable to junctional cancer [6].

Accumulating studies reveal that the molecular characteristics, pathological course, and clinical behavior of junctional cancer differ from that of gastric and esophageal cancer [7]. Junctional cancer primarily refers to adenocarcinoma of the esophagogastric junction (AEGJ). It is based on Siewert’s anatomical classification criteria and includes distal esophageal adenocarcinoma (EAC), cardiac cancer, and proximal gastric adenocarcinoma (GAC) [8]. The AEGJ incidence has risen rapidly in East Asia, North America, and Europe over the last few decades [9]. As the early symptoms are not obvious with a rapid progression, AEGJ is usually diagnosed in the late stages and has a 5-year survival rate of ~6% in the developing world [10]. Therefore, there is an urgent need to identify potential molecular markers to predict and improve the prognosis.

A specific component of the tumor immune microenvironment (TME), tumor-infiltrating lymphocytes (TILs) are reflective of host-tumor immune interactions and are predictive of patient prognosis [11, 12]. TILs primarily include T and B cells, and natural killer (NK) cells, which cooperate with tumor cells by releasing chemokines and cytokines that act as important tumorigenic and prognostic factors and determine tumor progression and aggressiveness [13]. Different cancer types have distinct TME, where numerous clinical studies that evaluated the TIL content in breast carcinoma, colorectal carcinoma, and non-small cell lung carcinoma reported that higher TIL infiltration conferred a significant survival benefit [14–16]. Besides, the observation of the variations in TILs levels can recognize the population or cancer types with a high likelihood of reacting to immunotherapy [13]. However, there are few studies focusing on the characteristics of tumor-infiltrating lymphocytes (TILs) in esophagogastric tumors and their potential as prognostic markers to predict and improve survival in AEGJ patients. Additionally, the association between TILs and survival in esophageal squamous cell carcinoma (ESCC) and GAC remains controversial [17, 18].

Although the gold standard for evaluating TILs is based on routine haematoxylin–eosin (H&E) staining using a semi-quantitative scoring method, it may be subject to interobserver variability and costly [19]. Computational pathology has currently displayed promise in recognizing the biomarkers in tissues, and overcomes limitations related with manual grading and human bias [20]. Therefore, we establish cellular training sets for AEGJ, GAC, and ESCC based on the assessment of experienced pathologists. Then, quantification of the TILs on H&E staining sections using an open-source image processing tool that operates with minimal user intervention. We compared the AEGJ TILs characteristics with that of GAC and ESCC under demographic factors and clinical traits. We also examined the association of auto-assessed TILs as a quantitative variable with overall survival in both large datasets.

## RESULTS

### Demographic information

Table 1 displays the demographic information of the 214 AEGJ, 256 GAC, and 752 ESCC cases included in the analysis. There were significant differences between AEGJ, GAC, and ESCC for age, sex, tea drinking, wealth score, first-line treatment method, TNM staging, tumor differentiation grade, *Helicobacter pylori (HP)* infection status, and gastric atrophy (all *P* < 0.05). Compared with ESCC and GAC, AEGJ cases were more likely to be older (mean age: 69.23 years), drink less tea (77.10%), have positive HP status (78.50%), receive combination therapy (24.30%), have advanced TNM stage (28.97%), and have gastric atrophy (25.23%).

**Table 1:** Demographic information of 214 AEGJ, 256 GAC, and 752 ESCC cases in Taixing (2010–2014).

**Variables**	**AEGJ (*n* = 214), *N* (%)**	**GAC (*n* = 256), *N* (%)**	**ESCC (*n* = 752), *N* (%)**	***P*-value**
**Age, mean ± SD, years**	69.23 (7.61)	67.52 (9.56)	66.96 (8.50)	0.002
**Age (years)**				
<60	22 (10.28)	57 (22.27)	140 (18.62)	0.002
≥60	192 (89.72)	199 (77.73)	612 (81.38	
**Sex**				0.027
Man	152 (71.03)	189 (73.83)	492 (65.43)	
Woman	62 (28.97)	67 (26.17)	260 (34.57)	
**Marriage**				**0.089**
Unmarried	12 (5.61)	9 (3.52)	25 (3.32)	
Married	153 (71.50)	186 (72.66)	591 (78.59)	
Divorce/widow	49 (22.90)	61 (23.83)	136 (18.09)	
**Educational level**				0.302
Illiteracy	74 (35.58)	80 (31.25)	276 (36.70)	
Primary or Secondary school	129 (60.28)	158 (61.72)	429 (57.05)	
High school and above	11 (5.14)	18 (7.03)	47 (6.25)	
**Cigarette smoking**				0.820
Never	86 (40.19)	103 (40.23)	313 (41.62)	
Ever or still	120 (56.07)	144 (56.25)	405 (53.86)	
Missing	8 (3.74)	9 (3.52)	34 (4.52)	
**Alcohol drinking**				**0.075**
Never	121 (56.54)	125 (48.83)	351 (46.68)	
Ever or still	88 (41.12)	122 (47.66)	366 (48.67)	
Missing	5 (2.34)	9 (3.52)	35 (4.65)	
**Tea drinking**				0.013
Never	165 (77.10)	170 (66.41)	493 (65.56)	
Ever	44 (20.56)	77 (30.08)	225 (29.92)	
Missing	5 (2.34)	9 (3.52)	34 (4.52)	
**Fruit intake (g/d)**				0.513
<25	110 (51.40)	136 (53.13)	417 (55.16)	
≥25	91 (42.52)	102 (39.84)	288 (38.10)	
Missing	13 (6.07)	18 (7.03)	47 (6.22)	
**Pickles intake (g/d)**				0.914
<10	124 (57.94)	141 (55.08)	419 (55.72)	
≥10	80 (37.38)	98 (38.28)	288 (38.30)	
Missing	10 (4.67)	17 (6.64)	45 (5.98)	
**BMI**				0.350
<18.5	27 (12.62)	32 (12.50)	85 (11.30)	
18.5–24	140 (65.42)	158 (61.72)	466 (61.97)	
≥24	46 (21.50)	66 (25.78)	201 (26.73)	
Missing	1 (0.47)	0 (0.00)	0 (0.00)	
**Wealth scores**				0.003
Q1	62 (28.97)	52 (20.31)	232 (30.85)	
Q2	45 (21.03)	55 (21.48)	139 (18.48)	
Q3	38 (17.76)	62 (24.42)	161 (21.41)	
Q4	43 (20.09)	47 (18.36)	138 (18.35)	
Q5	26 (12.15)	40 (15.63)	62 (8.24)	
**First-line treatment method**				<0.001
Radiotherapy	9 (4.21)	9 (3.52)	88 (11.70)	
Chemotherapy	21 (9.81)	27 (10.55)	93 (12.37)	
Surgery	116 (54.21)	151 (58.98)	377 (50.13)	
Combination therapy	52 (24.30)	57 (22.27)	170 (22.61)	
Untreated	14 (6.54)	11 (4.30)	21 (2.79)	
Missing	2 (0.93)	1 (0.39)	3 (0.40)	
**TNM staging**				0.004
0+I+II	55 (25.70)	98 (38.28)	256 (30.04)	
III+IV	62 (28.97)	50 (19.53)	162 (21.54)	
Missing	97 (45.33)	108 (42.19)	334 (44.41)	
**Grade of differentiation**				0.036
Gx Grading cannot be evaluated	36 (16.82)	47 (18.36)	40 (5.32)	
G1 Highly differentiated	4 (0.53)	7 (2.73)	60 (7.98)	
G2 Medium differentiation	63 (29.44)	63 (24.61)	452 (60.11)	
G3 Poorly differentiated	29 (13.55)	48 (18.75)	134 (17.82)	
G4 Undifferentiated	11 (5.14)	9 (3.52)	45 (5.98)	
Missing	71 (33.18)	82 (32.03)	21 (2.79)	
***Helicobacter pylori***				0.001
HP+	168 (78.50)	181 (70.70)	484 (64.36)	
HP−	42 (19.63)	68 (26.56)	234 (31.12)	
Missing	4 (1.87)	7 (2.73)	34 (4.52)	
**Gastric atrophy**				<0.001
Yes	54 (25.23)	51 (19.92)	98 (13.03)	
No	131 (61.21)	157 (61.33)	520 (69.15)	
Missing	29 (13.55)	157 (61.33)	134 (17.82)	

### Automated cellular recognition accuracy

**Figure 1:**
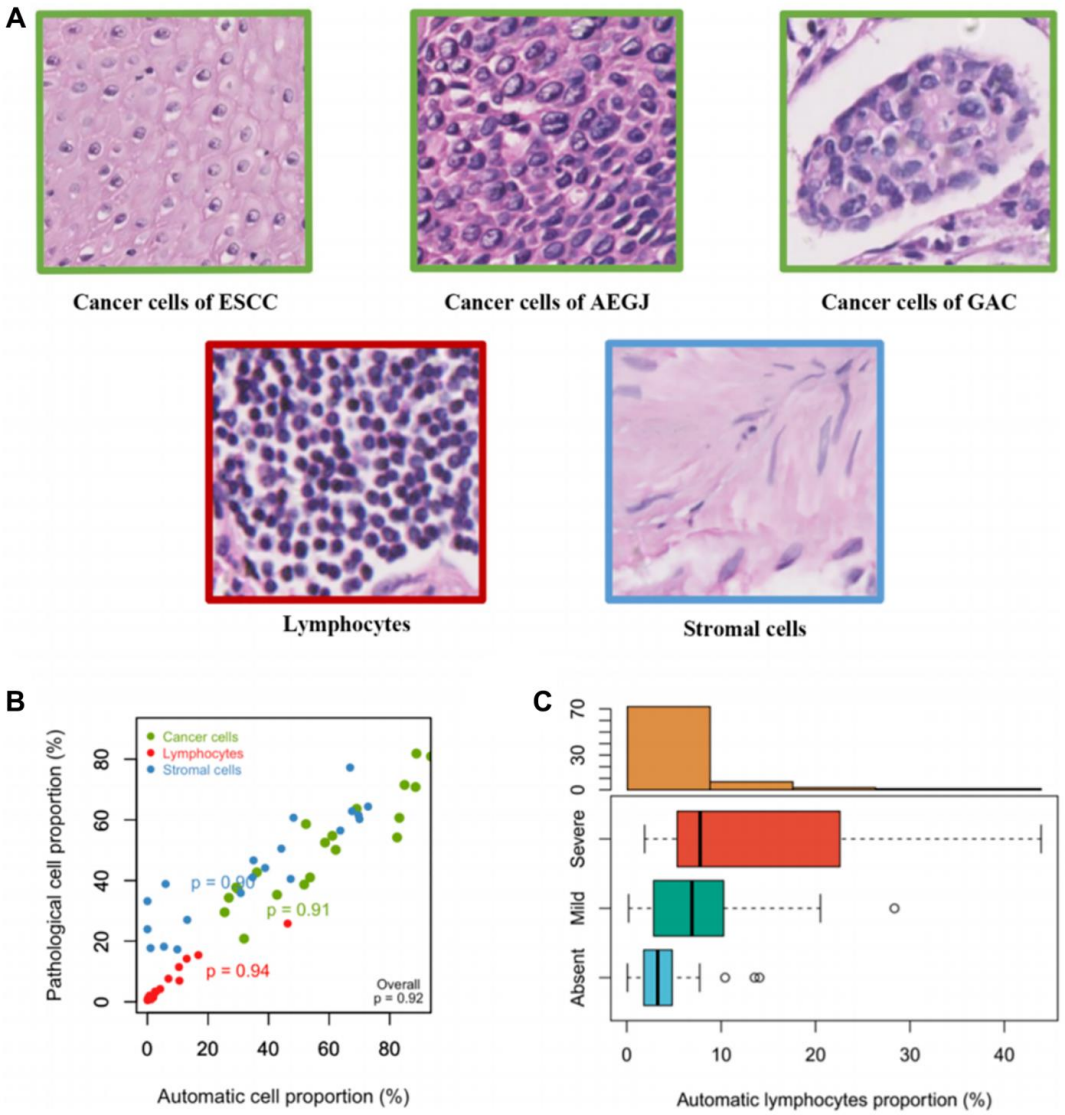
**Establishment and verifications of the cell training set in AEGJ.** (**A**) Example images of the three classes used in the classifier: cancer cells, lymphocytes, and stromal cells. (**B**) Cell proportions obtained by automated image analysis were compared to pathologists’ counts for a total of 10,000 single cells in a representative set of 20 tissue samples within AEGJ. (**C**) TILs proportions versus manual grading for AEGJ TIL infiltration in random one-third samples.

We obtained the matched H&E-stained images of 214 AEGJ, 256 GAC, and 752 ESCC cases, each of which was from a solid tumor cross-section. The image processing approach automatically segmented the images and classified the cellular components into cancer cells, lymphocytes, and stromal cells. The classification was based on cellular training sets using an SVM classifier that pathologists had trained according to the cell features (Figure 1A). Cross-validation within the cellular training sets of the three cancers yielded overall classification accuracy of >87.55% (Supplementary Table 1). Furthermore, the overall correlation coefficients between automated recognition and the pathologists’ quantitative assessment of AEGJ, GAC, and ESCC were 0.92, 0.93, and 0.93, respectively, and the TILs correlation coefficients were 0.94, 0.95, and 0.95, respectively. The correlation coefficients of the cancer cells and stromal cells were all >0.86 (Figure 1B; Supplementary Figure 1A, 1B). Furthermore, the automated recognition of the TILs proportion in AEGJ, GAC, and ESCC was consistent with the manual grading, and all Jonckheere-Terpstra tests yielded *P* = 0.001 (Figure 1C; Supplementary Figure 1C, 1D). The AEGJ, GAC, and ESCC cellular training sets are provided in the Supplementary Datasets 1–3.

### The auto-quantified TILs characteristics in AEGJ, GAC, and ESCC

**Figure 2:**
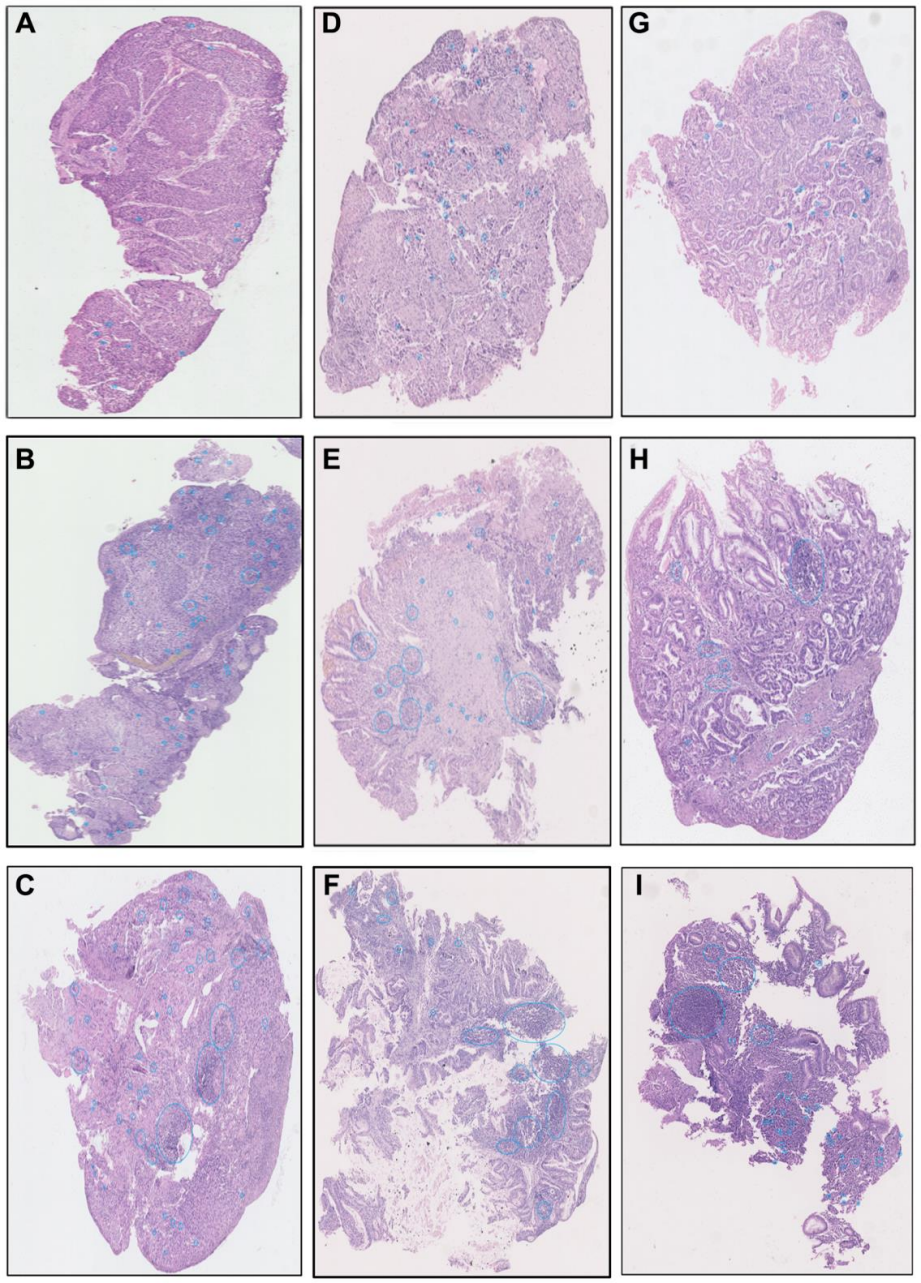
The examples images of low, medium, and high TILs intensity in ESCC (**A**–**C**), AEGJ (**D**–**F**), and GAC (**G**–**I**) H&E-stained tissue sections (H&E×200).

Based on the cellular training sets, we extracted the TILs proportions of 214 AEGJ, 256 GAC, and 752 ESCC cases. The examples of low, medium, and high TILs intensity circled by blue in ESCC, AEGJ, and GAC H&E-stained tissue sections and their raw images were displayed (Figure 2A–2I and Supplementary Figure 2A–2I). The association between TILs proportion and demographic information of the above three gastroesophageal cancers was listed in Table 2. In the AEGJ cases, the TILs percentage was associated with the first-line treatment method (*P* = 0.030), and the patients with the combination therapy (median TILs: 5.72%) had the highest TILs proportion compared to radiotherapy (median TILs: 1.83%), chemotherapy (median TILs: 4.10%), and surgery (median TILs: 2.77%). A similar association between TILs and the first-line treatment method had also discovered in GAC patients (*P* = 0.031). Besides, the GAC patients drinking more tea (median TILs: 2.74%) were more likely to have a higher TILs proportion (*P* = 0.028). In the ESCC cases, the patients eating fewer pickles (median TILs: 0.15%) were more likely to have a higher TILs level (*P* = 0.002), and the trend tests demonstrated the TILs proportion increased with the BMI ranks (*P* = 0.008).

**Table 2:** The association between TILs proportion and demographic information of AEGJ, GAC, and ESCC cases in Taixing.

**Variables**	**TILs (%) in AEGJ (*n* = 214)**		**TILs (%) in GAC (*n* = 256)**		**TILs (%) in ESCC (*n* = 752)**	
	**Median (IQR)**	***P*-value**	**Median (IQR)**	***P*-value**	**Median (IQR)**	***P*-value**
**Age (years)**		0.775		0.711		0.090
<60	3.82 (1.57, 11.05)		1.86 (0.16, 9.16)		0.15 (0.03, 0.92)	
≥60	4.88 (2.01, 11.21)		1.93 (0.47, 8.16)		0.12 (0.02, 0.73)	
**Sex**		0.479		**0.096**		0.437
Man	4.55 (1.71, 10.80)		2.38 (0.52, 9.14)		0.12 (0.02, 0.67)	
Woman	5.19 (2.41, 12.45)		1.27 (0.30, 6.54)		0.13 (0.02, 1.10)	
**Marriage**		0.779		0.702		0.295
Unmarried	8.02 (1.91, 11.32)		1.14 (0.43, 1.81)		0.11 (0.03, 0.43)	
Married	4.60 (1.86, 10.42)		2.36 (0.41, 9.30)		0.12 (0.02, 0.75)	
Divorce/widow	5.19 (2.52, 13.93)		2.18 (0.52, 3.55)		0.17 (0.03, 0.76)	
**Educational level**		0.455		0.108		0.868
Illiteracy	5.19 (2.58, 11.06)		1.16 (0.35, 4.48)		0.11 (0.02, 0.79)	
Primary or Secondary school	4.65 (1.66, 11.30)		2.50 (0.70, 10.43)		0.13 (0.02, 0.74)	
High school and above	3.23 (1.09, 7.31)		3.09 (0.11, 10.14)		0.12 (0.02, 0.92)	
**Cigarette smoking**		0.683		**0.025**		0.173
Never	4.34 (1.35, 13.89)		1.16 (0.21, 7.54)		0.13 (0.02, 1.06)	
Ever or still	4.95 (2.38, 10.50)		2.49 (0.72, 9.43)		0.11 (0.02, 0.63)	
Missing	NA		NA		NA	
**Alcohol drinking**		0.357		0.432		0.252
Never	5.42 (1.86, 13.35)		1.59 (0.36, 7.99)		0.13 (0.02, 0.91)	
Ever or still	4.17 (2.20, 9.41)		2.38 (0.51, 8.99)		0.11 (0.02, 0.64)	
Missing	NA		NA		NA	
**Tea drinking**		0.271		0.028		0.997
Never	4.65 (1.56, 11.01)		1.51 (0.29, 7.93)		0.12 (0.02, 0.72)	
Ever	5.36 (3.02, 11.11)		2.74 (1.14, 10.47)		0.12 (0.02, 0.75)	
Missing	NA		NA		NA	
**Fruit intake (g/d)**		0.613		0.788		0.623
<25	4.30 (2.07, 10.41)		2.23 (0.47, 7.80)		0.13 (0.02, 0.79)	
≥25	5.23 (1.51, 12.34)		1.90 (0.37, 9.58)		0.10 (0.02, 0.67)	
Missing	NA		NA		NA	
**Pickles intake (g/d)**		0.091		0.164		**0.002**
<10	5.12 (1.63, 16.54)		2.40 (0.51, 9.14)		0.15 (0.03, 0.96)	
≥10	3.95 (2.29, 9.66)		1.39 (0.36, 6.21)		0.08 (0.02, 0.47)	
Missing	NA		NA		NA	
**BMI**		0.737		0.700		**0.039**
<18.5	5.02 (1.32, 8.99)		1.83 (0.33, 6.88)		0.08 (0.02, 0.56)	
18.5–24	4.56 (1.99, 11.21)		2.34 (0.51, 8.08)		0.11 (0.02, 0.62)	
≥24	4.97 (2.09, 10.82)		1.86 (0.43, 9.15)		0.23 (0.02, 1.20)	
Missing	NA		NA		NA	
**Wealth scores**		0.124		0.707		0.085
Q1	4.99 (2.47, 11.56)		2.13 (0.37, 8.91)		0.12 (0.02, 0.69)	
Q2	2.94 (1.36, 5.60)		1.27 (0.36, 4.56)		0.13 (0.02, 0.96)	
Q3	5.22 (2.24, 13.93)		2.55 (0.38, 12.08)		0.09 (0.01, 0.45)	
Q4	4.26 (1.23, 10.69)		2.38 (0.67, 10.67)		0.13 (0.02, 0.51)	
Q5	5.71 (3.41, 13.55)		2.41 (0.93, 4.74)		0.25 (0.03, 2.05)	
**First-line treatment method**		0.030		0.031		0.262
Radiotherapy	1.83 (1.05, 3.49)		0.66 (0.18, 1.61)		0.03 (0.02, 0.43)	
Chemotherapy	4.10 (1.56, 5.30)		1.32 (0.30, 13.71)		0.10 (0.02, 0.48)	
Surgery	2.77 (0.40, 7.72)		0.82 (0.27, 2.74)		0.09 (0.02, 0.68)	
Combination therapy	5.72 (3.14, 11.94)		2.78 (0.73, 11.12)		0.15 (0.03, 0.80)	
Untreated	4.64 (1.81, 10.42)		1.32 (0.35, 3.21)		0.10 (0.02, 0.92)	
Missing	NA		NA		NA	
**TNM staging**		0.750		0.841		0.996
0+I+II	6.92 (3.12, 13.39)		2.64 (0.49, 10.91)		0.14 (0.02, 0.76)	
III+IV	5.34 (2.99, 11.56)		2.47 (0.74, 11.12)		0.15 (0.02, 0.92)	
Missing	NA		NA		NA	
**Grade of differentiation**		0.207		0.930		0.954
Gx Grading cannot be evaluated	4.04 (1.77, 12.42)		2.38 (0.82, 10.20)		0.16 (0.02, 1.30)	
G1 Highly differentiated	8.67 (2.55, 14.28)		1.60 (0.68, 10.19)		0.13 (0.03, 0.69)	
G2 Medium differentiation	7.72 (3.89, 13.39)		2.78 (1.13, 9.15)		0.13 (0.02, 0.84)	
G3 Poorly differentiated	7.17 (1.97, 12.81)		2.10 (0.26, 11.47)		0.11 (0.02, 0.56)	
G4 Undifferentiated	4.34 (2.42, 5.17)		0.85 (0.35, 3.50)		0.13 (0.05, 0.35)	
Missing	NA		NA		NA	
***Helicobacter pylori***		0.599		0.721		0.707
HP+	5.24 (2.99, 11.10)		1.83 (0.39, 6.87)		0.12 (0.02, 0.60)	
HP−	4.62 (1.84, 11.24)		2.28 (0.45, 9.66)		0.13 (0.02, 0.77)	
Missing	NA		NA		NA	
**Gastric atrophy**		0.475		0.167		0.589
Yes	4.96 (1.92, 10.58)		2.40 (0.73, 10.68)		0.12 (0.02, 0.73)	
No	4.34 (1.46, 11.06)		1.31 (0.36, 6.32)		0.09 (0.02, 0.42)	
Missing	NA		NA		NA	

### The distribution of auto-quantified TILs proportion

We performed a crude comparison of the AEGJ TILs proportion with that of ESCC and GAC by The Kruskal-Wallis H test. The differences in the TILs proportions were statistically significant (*P* < 0.001). The multiple comparisons corrected by the Bonferroni method found that the AEGJ cases had the highest TILs proportion (median, 4.82%), followed by GAC (median, 1.92%), and that of ESCC was the lowest (median, 0.12%) (Supplementary Figure 3). However, lymphocyte infiltration is associated with age, sex, BMI, TNM staging, tumor differentiation grade, and first-line treatment method [21–23]. Hence, based on the above factors and the demographic information that differed among the three cancers, we compared the TILs proportion between these three cancers within each factor. The result revealed that significantly different among the above cancers still existed (Supplementary Table 2; all *P* < 0.001). Moreover, the distribution of auto-quantified TILs proportion was tested with Spearman correlation analysis and multi-variable linear regression. After adding the cancer type as a variable to the analysis-adjusted covariates, cancer type remained associated with TILs proportion (Table 3; *ρ* = 0.49; *P* < 0.001). The standardised effects of AEGJ and GAC were 0.36 and 0.28, respectively (Table 3; R^2^ = 0.204; adjusted R^2^ = 0.179; *P* < 0.001). This indicated that the cancer type contributed considerably to explaining variations in the distribution of auto-assessed TILs proportion. We also determined that the cancer type is AEGJ had the most influence on TILs proportion compared with GAC and ESCC.

**Table 3:** Multi-variable linear regression analysis of the association between factors and TILs proportion.

**Variables**	**Correlation coefficient**	**Unstandardized coefficient (B)**		**Standardized coefficient (Beta)**	***P*-value**
		**B (95% CI)**	**SE**		
**Age**					
<60	0.01	Ref.			
≥60		−0.03 (−1.62, 1.57)	0.81	−0.01	0.975
**Sex**					
Woman	−0.03	Ref.			
Man		−0.06 (−1.54, 1.43)	0.75	0.00	0.942
**BMI**					
<18.5	0.04	Ref.			
18.5–24		0.36 (−1.72, 2.44)	1.06	0.02	0.734
≥24		0.50 (−1.77, 2.78)	1.16	0.03	0.664
***Helicobacter pylori***					
HP−	0.06	Ref.			
HP+		0.21 (−1.22, 1.63)	0.73	0.01	0.776
**Pickles intake**					
<10	0.04	Ref.			
≥10		−1.20 (−2.51, 0.11)	0.67	−0.07	0.07
**Tea drinking**					
Never	0.00	Ref.			
Ever		0.28 (−1.26, 1.83)	0.79	0.01	0.721
**First-line treatment method**					
Untreated	0.06	Ref.			
Radiotherapy		−13.94 (−22.20, −5.67)	4.21	−0.39	**0.001**
Chemotherapy		−14.42 (−22.58, −6.26)	4.15	−0.46	**0.001**
Surgery		−13.52 (−21.40, −5.63)	4.01	−0.74	**0.001**
Combination therapy		−13.85 (−21.81, −5.88)	4.06	−0.64	**0.001**
**Grade of differentiation**					
G1 Highly differentiated	−0.06	Ref.			
G2 Medium differentiation		−0.03 (−2.53, 2.47)	1.27	−0.00	0.981
G3 Poorly differentiated		0.51 (−2.35, 3.38)	1.46	0.02	0.725
G4 Undifferentiated		−1.46 (−4.87, 1.95)	1.74	−0.05	0.401
Gx Grading cannot be evaluated		1.21 (−2.39, 4.81)	1.83	0.03	0.510
**TNM staging**					
0+I+II	0.03	Ref.			
III+IV		0.21 (−1.24, 1.66)	0.74	0.01	0.776
**Cancer type**					
ESCC	0.49	Ref.			
GAC		6.07 (4.32, 7.82)	0.89	0.28	**<0.001**
AEGJ		8.28 (6.45, 10.12)	0.94	0.36	**<0.001**
Summary	R^2^ = 0.204		Adjusted R^2^ = 0.179		**<0.001**

### The prognostic value of auto-quantified TILs proportion in AEGJ, GAC, and ESCC

**Figure 3:**
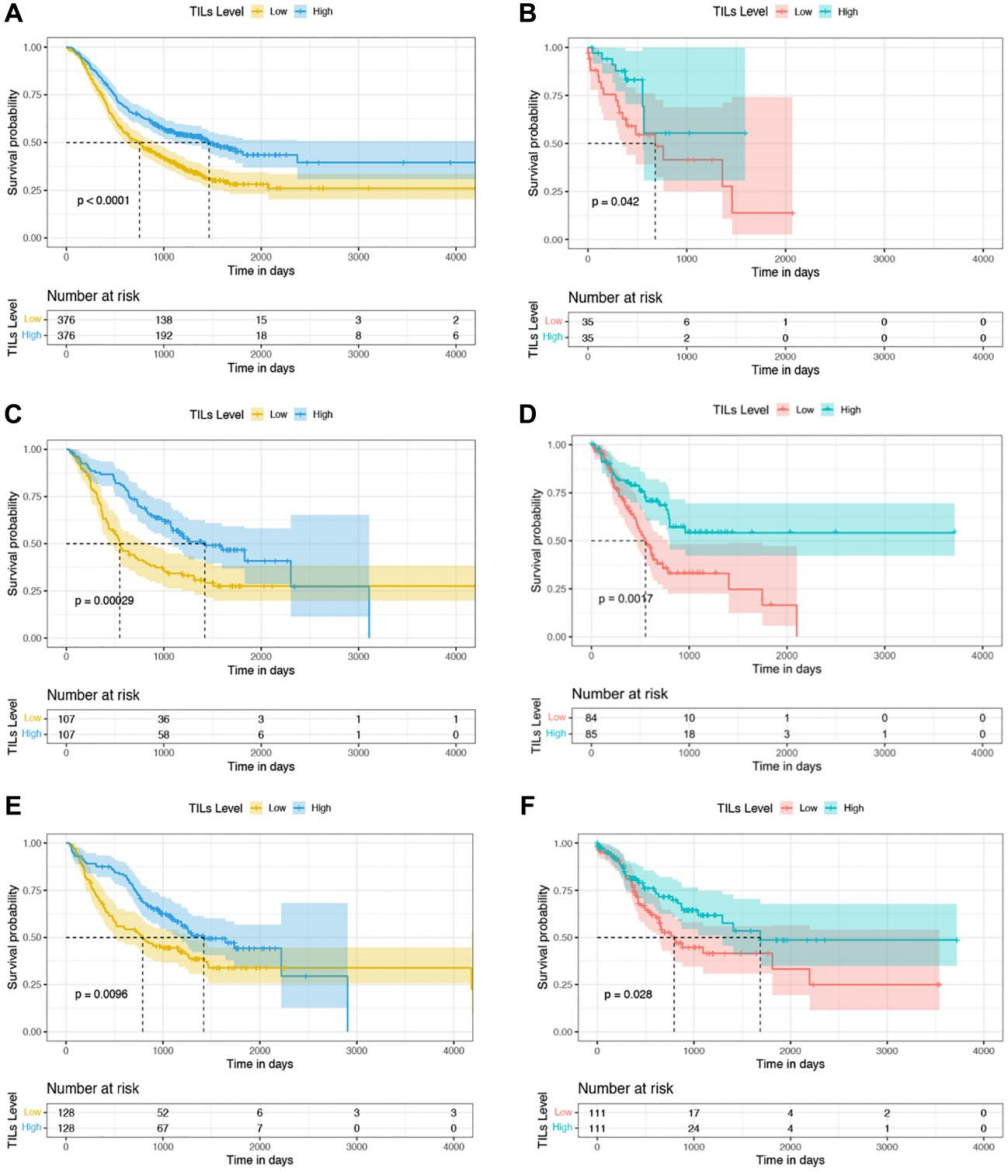
**Kaplan-Meier curves of OS based on TILs proportion in the discovery and validation datasets.** (**A**, **C**, **E**) Survival analysis between the high- and low-TILs groups in 752 ESCC, 214 AEGJ, and 256 GAC cases in Taixing, China, 2010–2014. (**B**, **D**, **F**) Survival analysis between the high- and low-TILs groups in 70 ESCC, 169 AEGJ, and 222 GAC cases of the TCGA dataset.

To explore whether there was a prognostic value of the auto-assessed TILs proportion in AEGJ, GAC, and ESCC, we utilized the cases from Taixing as discovery (Taixing: 214 AEGJ, 256 GAC, and 752 ESCC cases) and these of TCGA as validation (TCGA: 169 AEGJ, 222 GAC, and 70 ESCC cases). The AEGJ, GAC, and ESCC cases from the Taixing dataset were divided into high- and low-TIL groups using median TILs proportions of 4.82%, 1.92%, and 0.12% as cut-offs, respectively. The AEGJ, GAC, and ESCC cases from the TCGA dataset were divided using median TILs proportions of 1.99%, 4.14%, and 32.40%, respectively, as cut-offs, and evaluated the prognostic value in both datasets. Kaplan-Meier plots were generated to compare the OS based on high and low TILs proportions. There were statistically significant associations between better OS and higher TILs proportion in the three cancers of the Taixing dataset (Figure 3A, 3C, 3E; all *P* < 0.001). As validation, we also identified statistically significant differences in OS between the high- and low-groups of the TCGA dataset (Figure 3B, 3D, 3F; all *P* < 0.05).

We further examined the independent prognostic value of the auto-quantified TILs proportion. A multivariable Cox regression analysis was performed on the discovery and validation. In discovery, the marriage and TILs proportion were statistically related to the outcome in AEGJ cases (Table 4). Education level, first-line treatment method, gastric atrophy, and TILs proportion were significantly associated with prognosis in GAC cases (Supplementary Table 3). The age, sex, marriage, first-line treatment method, differentiation grade, and TILs proportion were significantly associated with OS in the ESCC cases (Supplementary Table 4). Specifically, a higher TIL percentage was associated with a better prognosis in AEGJ (adjusted HR (aHR) = 0.965; 95% CI = 0.938–0.994; *P* = 0.017), GAC (aHR = 0.972; 95% CI = 0.949–0.995; *P* = 0.016), and ESCC (aHR = 0.967; 95% CI = 0.938–0.997; *P* = 0.032) cases (Table 4 and Supplementary Tables 3 and 4). Notably, after further adjusting for TNM staging and other covariates, the associations between TILs proportion and OS were still significant in the AEGJ (aHR = 0.946; 95% CI = 0.907–0.986; *P* = 0.009), GAC (aHR = 0.961; 95% CI = 0.931–0.993; *P* = 0.016), and ESCC (aHR = 0.954; 95% CI = 0.911–0.998; *P* = 0.041) cases (Supplementary Tables 5–7). In validation, except the ESCC (aHR = 0.984; 95% CI = 0.965–1.005; *P* = 0.133) cases, the auto-quantified TILs also were an independent prognostic biomarker in AEGJ (aHR = 0.812; 95% CI = 0.712–0.925; *P* = 0.002), GAC (aHR = 0.969; 95% CI = 0.942–0.996; *P* = 0.025) cases (Supplementary Tables 8–10).

**Table 4:** Univariable and multivariable Cox regression analyses of basic characteristics with OS in AEGJ of Taixing dataset (Discovery, *N* = 214).

**Characteristics**	**No. of patients (%)**	**Median survival (years)**	**Univariable analysis**		**Multivariable analysis**	
			***P*-value**	**HR (95% CI)**	***P*-value**	**aHR^a^ (95% CI)**
**TILs, Median (IQR)**	4.82 (1.90, 11.18)		**<0.001**	0.964 (0.943, 0.985)	**0.017**	0.965 (0.938, 0.994)
**Age, mean (range)**	69.23 (49–84)		0.488	1.008 (0.986, 1.031)	0.849	1.003 (0.970, 1.037)
**Sex**						
Woman	62 (28.97)	2.44		Ref.		Ref.
Man	152 (71.03)	2.32	0.640	1.097 (0.743, 1.621)	0.852	0.950 (0.552, 1.634)
**Marriage**						
Divorce/widow	49 (22.90)	2.51		Ref.		Ref.
Unmarried	12 (5.61)	2.71	0.286	0.597 (0.231, 1.540)	**0.019**	0.125 (0.022, 0.708)
Married	153 (71.50)	2.26	0.819	0.953 (0.630, 1.441)	0.383	0.749 (0.391, 1.434)
**Educational level**						
High school and above	11 (5.14)	1.95		Ref.		Ref.
Primary or Secondary school	129 (60.28)	2.39	0.175	0.633 (0.327, 1.226)	0.253	1.881 (0.637, 5.550)
Illiteracy	74 (35.58)	2.52	0.130	0.586 (0.293, 1.171)	0.496	1.258 (0.650, 2.435)
**Cigarette smoking**						
Never	313 (41.62)	2.48		Ref.		Ref.
Ever or still	120 (56.07)	2.45	0.813	0.957 (0.666, 1.375)	0.927	1.023 (0.622, 1.683)
Missing	8 (3.74)					
**Alcohol drinking**						
Never	121 (56.54)	2.39		Ref.		Ref.
Ever or still	88 (41.12)	2.44	0.979	1.005 (0.701, 1.441)	0.767	0.930 (0.574, 1.505)
Missing	5 (2.34)					
**Tea drinking**						
Never	165 (77.10)	2.51		Ref.		Ref.
Ever	44 (20.56)	2.14	0.930	1.020 (0.656, 1.585)	0.909	1.033 (0.590, 1.809)
Missing	5 (2.34)					
**BMI**						
≥24	46 (21.50)	2.57		Ref.		Ref.
18.5–24	140 (65.42)	2.45	0.587	0.890 (0.585, 1.354)	0.706	0.893 (0.497, 1.607)
<18.5	27 (12.62)	1.68	0.611	1.171 (0.638, 2.151)	0.800	1.116 (0.477, 2.608)
Missing	1 (0.47)					
**Wealth scores**						
Q5	26 (12.15)	2.35		Ref.		Ref.
Q4	43 (20.09)	2.35	0.682	1.145 (0.600, 2.187)	0.647	0.817 (0.345, 1.939)
Q3	38 (17.76)	2.45	0.936	0.973 (0.494, 1.914)	0.916	0.952 (0.379, 2.390)
Q2	45 (21.03)	1.98	0.566	1.206 (0.636, 2.288)	0.457	0.718 (0.300, 1.719)
Q1	62 (28.97)	2.53	0.862	1.056 (0.570, 1.959)	0.430	0.714 (0.309, 1.647)
**First-line treatment method**
Untreated	14 (6.54)	1.44		Ref.		Ref.
Radiotherapy	9 (4.21)	0.99	0.294	0.598 (0.229, 1.563)	0.424	0.473 (0.075, 2.967)
Chemotherapy	21 (9.81)	1.19	0.256	0.664 (0.327, 1.347)	0.610	0.723 (0.208, 2.516)
Surgery	116 (54.21)	2.94	**<0.001**	0.235 (0.129, 0.426)	0.303	0.542 (0.169, 1.738)
Combination therapy	52 (24.30)	2.05	**0.002**	0.369 (0.196, 0.695)	0.469	0.637 (0.188, 2.162)
Missing	2 (0.93)					
**Grade of differentiation**
G1 Highly differentiated	4 (0.53)	2.19		Ref.		Ref.
G2 Medium differentiation	63 (29.44)	3.00	0.573	0.661 (0.156, 2.790)	0.625	0.695 (0.161, 2.994)
G3 Poorly differentiated	29 (13.55)	3.13	0.572	0.894 (0.204, 3.919)	0.926	0.931 (0.206, 4.203)
G4 Undifferentiated	11 (5.14)	0.68	**0.048**	4.665 (1.015, 21.443)	0.058	4.655 (0.947, 22.879)
Gx Grading cannot be evaluated	36 (16.82)	1.48	0.348	1.993 (0.473, 8.405)	0.396	1.886 (0.436, 8.156)
Missing	71 (33.18)					
***Helicobacter pylori***						
HP−	42 (19.63)	2.36		Ref.		Ref.
HP+	168 (78.50)	2.36	0.959	1.012 (0.654, 1.564)	0.794	1.083 (0.597, 1.962)
Missing	4 (1.87)					
**Gastric atrophy**						
No	131 (61.21)	2.26		Ref.		Ref.
Yes	54 (25.23)	2.13	0.116	1.362 (0.927, 2.001)	0.164	1.466 (0.855, 2.514)
Missing	29 (13.55)					

^a^aHR with adjustment for TILs proportion, age, sex, differentiation grade, first-line treatment method, and BMI.

## DISCUSSION

Although a standardized methodology for manual TILs assessment exists, it has several limitations due to requiring professional pathologists, interobserver variability, and higher costs. To address these problems, our study established the cellular training sets, respectively, explored the characteristics of auto-quantified TILs in AEGJ, GAC, and ESCC. The prognostic value of auto-assessed TILs was investigated in the above esophagogastric tumors. The TILs proportion was distinctive between different demographic and clinical traits and was the highest in AEGJ compared with GAC and ESCC in Taixing. The auto-quantified TILs were an independent prognostic biomarker for AEGJ, GAC, and ESCC.

The characteristics of TILs infiltration are distinctive in different body mass, eating habits, and cancer treatment methods. The tumor microenvironment (TME) is a specific metabolic niche composed of various cellular components as well as the contents of the tumor interstitial space. Recent research data have revealed that high-fat diet-induced obesity contributes to the tumor cell fat uptake, whereas the CD8+ T cell intaking the energy was suppressed [24]. These distinctive adaptations impaired the lymphocyte infiltration degree. However, our study showed the TILs proportion tends to enrich with increasing BMI ranks. This may be related to the types of TILs contained. The TILs include diverse immune cells, e.g., T cells, B cells, and NK cells [25]. In this research, we regard TILs as a major category that might weaken the above association. Our study also discovered that patients drinking more tea and intaking fewer pickles were more likely to enrich the TILs. These results were consistent with previous experiments. Mantena et al. [26] proved that the tea polyphenols that originated from tea contributed to the increasing recruitment of TILs in TME. Besides, eating excessive pickles leads to more nitrite intake, which easily oxidizes hemoglobin to methemoglobin, resulting in the lower oxygen-carrying capacity of blood and facilitating the formation of an immunosuppressive environment [27]. Additionally, the patients with combination therapy had the most TILs levels compared to radiotherapy, chemotherapy, and surgery in our cases. These results support that combination therapy produces fewer side effects on immune cells than applying the above three methods alone [28]. Hence, keeping a better diet and body quality, and choosing a suitable cancer therapy in the clinical field will help to improve immune infiltration to resist tumor growth.

The TILs infiltration showed a cancer-specificity in esophagogastric cancers. Quantification of TILs is growing in significance as evidence emerges of a reliable biomarker to reflect the better response to immunotherapeutic agents [29]. Characterizing the TIL proportion between different solid tumors would provide clues into the varied effectiveness of in immunotherapy. In the general clinical field, the AEGJ, located between the esophagus and stomach is more likely to group with GAC. Nevertheless, increasing evidence demonstrated the AEGJ displayed a significant difference in immune molecular characteristics [30]. In this study, we revealed that the TILs proportion varied between esophagogastric cancer, where AEGJ had the highest TIL proportion. The absolute difference in the TILs proportions between AEGJ and GAC was smaller than that between ESCC and AEGJ or between ESCC and GAC. Our results were similar to previous studies. Mohamed et al. [31] studied 215 ESCC, 1176 EAC, and 1951 GAC cases, including gastric and gastroesophageal junction cancers, and reported that ESCC exhibited a unique molecular profile, whereas GAC and AEGJ shared similarities, supporting the idea that squamous cell carcinomas and adenocarcinomas are entirely different diseases at the molecular level. In another study of 4125 tumor specimens from patients with 14 different gastrointestinal cancer types, Alberto et al. demonstrated that AEGJ had different immune characteristics from GAC and EAC [32]. Our results supported and complemented these findings, indicating the specificity of AEGJ in lymphocyte infiltration degree compared to GAC and ESCC. In addition, variations of TILs infiltration in cancers also indirectly reflect the different immunotherapy effects. Our results might present evidence for the specific selection of immunotherapy for esophagogastric cancers. However, some trial examinations of PD-1/PD-L1 blockade in upper gastrointestinal cancers enrolled patients with gastric cancers and AEGJ without distinction [33]. Therefore, our results also provide clues for future clinical immunotherapy in esophagogastric cancers and enhance precision therapy.

The auto-assessed TIL proportion is an independent prognostic biomarker in AEGJ, GAC, and ESCC patients. The cumulative studies have focused on the association between semiquantitative scoring of TILs levels and prognosis in esophagogastric cancers, and high TILs scores have been reported as a positive prognosis marker [34–37]. Despite the standardized efforts, the subjective nature and higher costs have limited its translational adoption into clinical practice [38]. Besides, the prognostic biomarkers for AEGJ are still under-explored. For this reason, we used the automatic algorithm to quantify the TILs percentage and investigate its prognostic value. We performed a survival analysis of AEGJ and determined that the auto-quantified TILs proportion was an independent prognostic biomarker in Taixing and TCGA datasets. This finding was consistent with the results of two previous studies [39, 40], in which the TILs proportion was estimated by pathologists. However, several researchers demonstrated that the prognostic value of TILs in GAC and ESCC has not been defined [41–43]. Nevertheless, some studies also reported positive results [44–47]. In the present study, we also identified an association between higher auto-assessed TILs proportion and better overall survival in GAC and ESCC cases in both datasets. This discovery supported the idea of the prognostic value of TILs proportion in GAC and ESCC. Hence, we can predict the overall survival of AEGJ, GAC, and ESCC by auto-quantified TILs infiltration degree objectively and it has the potential for translation to the routine clinical and pathological application at minimal additional cost.

As far as we know, this is the first relatively comprehensive establishment of cellular training sets for esophagogastric tumors to automatically quantify TILs infiltration in AEGJ, GAC, and ESCC. We obtained relatively full demographic characteristics and clinical information to explore the TILs characteristics. The findings contributed to more accurate tumor classification and immunotherapy outcome prediction. As an independent prognostic factor common to AEGJ, the auto-quantified TILs provided more evidence for its predictive value in upper gastrointestinal tumors. The findings established the foundation for further exploration of TME differences at specific immune cell level, providing crucial insights into immunotherapy and supporting the prognostic value of the auto-quantified TILs proportion in esophagogastric tumors, particularly in the AEGJ patients.

This study has some limitations. Our study aims to provide clues for immunotherapy in patients with upper gastrointestinal tumors by comparing the TILs level as the immune characteristic. Although the cases in our study did not receive immunotherapy to directly draw relevant conclusions, our results can provide data support for immunotherapy in AEGJ to some extent. Besides, multivariable survival analysis in the validation set showed that auto-quantified TILs were not an independent prognostic factor for ESCC, which may be related to the small sample size of ESCC in TCGA. However, considering the large sample size of ESCC in the Taixing dataset, we can still consider the independent prognostic value of auto-quantified TILs in ESCC.

Future research should investigate the association between the auto-quantified TILs proportion and clinical outcomes in patients received immunotherapy. Moreover, incorporating additional independent datasets to deeply validate the independent prognostic value of TILs in patients with AEGJ, GAC and ESCC will enhance the value of the clinical application of this biomarker.

## CONCLUSIONS

The findings of this study suggested that TILs levels determined by CRImage based on three different cell training sets are showing distinctive characteristics between various demographic information, clinical traits, and cancer types. The auto-quantified TILs are an independent prognostic factor in AEGJ, GAC, and ESCC patients, and are associated with a favorable prognosis. It is a cost-effective biomarker to predict and improve prognosis in clinical and pathological research.

## MATERIALS AND METHODS

### Patients and sample selection

**Figure 4:**
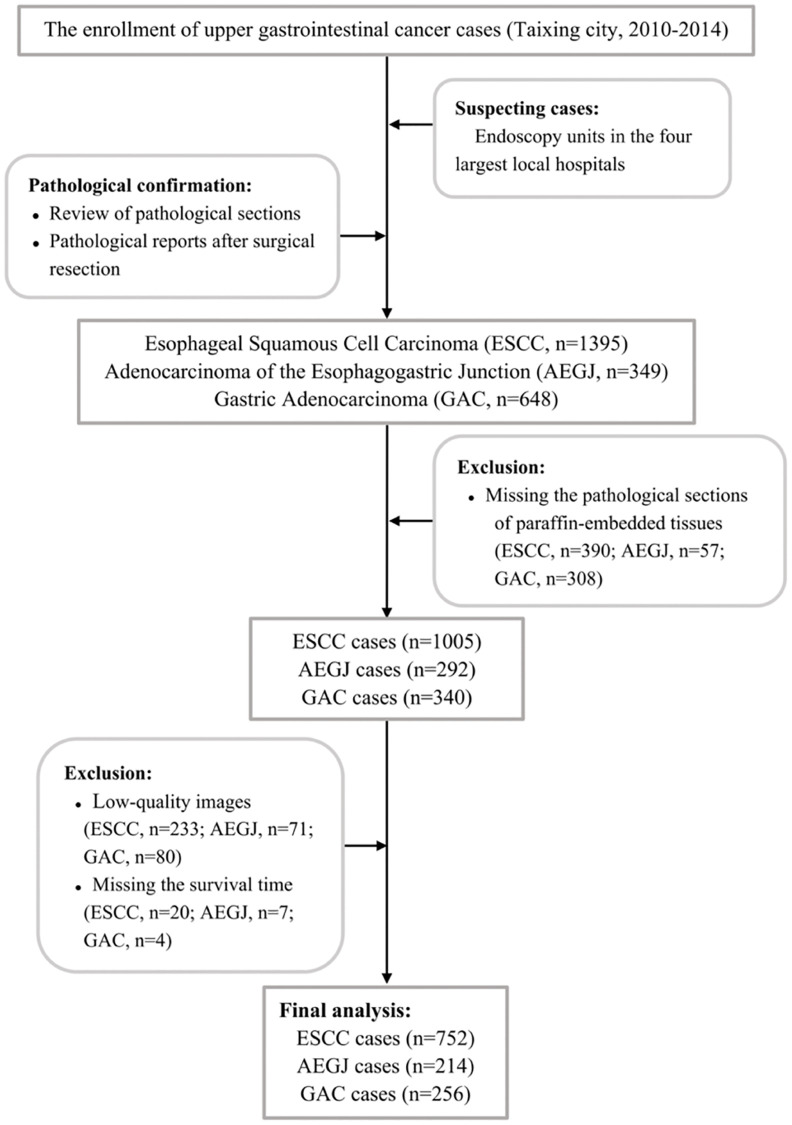
The flowchart of inclusion and exclusion of ESCC, AEGJ, and GAC cases in Taixing, Jiangsu (2010–2014).

The analysis was based on a 2010–2014 population-based case-control study in Taixing, Jiangsu, China, where there is a high incidence of upper gastrointestinal cancer. Patients were mainly recruited from the endoscopy units of the four most prominent hospitals in Taixing, the People’s Hospital of Taixing, the Second People’s Hospital of Taixing, the Third People’s Hospital of Taixing, and the Hospital of Traditional Chinese Medicine of Taixing. Patients with suspected ESCC, AEGJ, and GAC on endoscopy were invited to participate. The demographic information of patients was obtained using questionnaires and pathological sections of formalin-fixed, paraffin-embedded tissue blocks were obtained from the pathology department. The following inclusion criteria of the participants were applied: (1) age 40–85 years old and living in Taixing for >5 years; (2) suspected ESCC, AEGJ, and GAC by endoscopy that was subsequently pathologically confirmed; (3) preserved H&E staining tumor section images. The detailed research designs were described previously [48–50]. Based on the above criteria, the 1005 ESCC, 292 AEGJ, and 340 GAC cases were included. We also excluded 233 ESCC (23.18%), 71 AEGJ (24.32%), and 80 GAC (23.53%) cases with low-quality images due to uneven H&E-stained, unflattening, and blurred images. And the 20 ESCC (1.99%), 7 AEGJ (2.40%), and 4 GAC (1.18%) patients who missed the survival time were excluded. Finally, the 752 ESCC, 214 AEGJ, and 256 GAC cases were analyzed. Figure 4 illustrates the case selection flow. In the TCGA dataset, we excluded 34 AEGJ (16.75%), 51 GAC (18.68%), and 19 ESCC (21.35%) cases with low-quality images from the 203 AEGJ, 273 GAC, and 89 ESCC cases, respectively. The 169 AEGJ, 222 GAC, and 70 ESCC cases from the TCGA dataset were analyzed to validate the prognostic value of auto-quantified TILs.

### Pathological image processing pipeline

We utilized the pathological image processing pipeline published in our previous study [51]. The images of the H&E-stained tumor sections were processed using the R package CRImage developed by Yuan et al. [52]. Based on watershed segmentation and Otsu thresholding for haematoxylin-positive nuclei, this tool was embedded with the EBImage R package and a support vector machine (SVM) [52], to achieve color transformation and segmentation of the nuclei, then analyzed each morphological feature of each nuclei detected, such as shape, intensity, and texture features. The resulting morphological and textural features were input into the SVM for the supervised classification of cancer cells, lymphocytes, and stromal cells. The cancer cells exhibited large nuclei and variable texture and shape, and lymphocytes were small, round, and contained basophilic nuclei. Therefore, cancer cells and lymphocytes could be reliably differentiated from stromal cells that contained the elongated nuclei of fibroblasts and endothelial cells.

We selected the regions containing tumor cells, lymphocytes, and stromal cells from the tissue images and imported them into EBImage for conversion to the LAB color space. The mean and standard deviation of each channel were computed to convert the image to grayscale for further segmentation and cell recognition. Subsequently, an Otsu threshold to partition the image into foreground and background was constructed by the algorithm of maximization of the between-class variance method and morphological opening. Leveraging both the image grayscale and the threshold, the algorithm can eliminate noise and refine the cell edges. Eventually, the watershed segmentation was performed to separate cell clusters, automatically outlining recognized cells in the image.

The senior pathologists were invited to discern the circled cell types, identifying them as cancer cells, lymphocytes, and stromal cells. Subsequently, the EBImage toolkit integrated within CRImage was employed to extract 43 cellular features, encompassing nucleus perimeter, major axis, eccentricity, and the count of neighboring cells, among additional metrics. These features were then exported to construct training sets comprising cellular characteristics for each cancer type. Considering the cytomorphological differences between AEGJ, GAC, and ESCC, we established cell training sets for each cancer. We performed three verifications to test the accuracy of the image analysis tool based on our training sets: (1) cross-validation within each training set; (2) correlation between 10,000 single-cell annotations by the pathologists and automated recognition; (3) comparison of auto-quantified TILs proportion with manual infiltration grade of TILs evaluated by expert pathologists in random one-third samples of each cancer type.

We enhanced the computational efficiency of the algorithm by dividing each entire H&E-stained tumor section image into 100 equal parts. The established AEGJ, GAC, and ESCC cell feature training sets were loaded into the SVM embedded in CRImage to create cell classifiers, respectively. Utilizing the corresponding classifier to read the target H&E-stained tumor tissue sections, we obtained the cell counts and measured lymphocyte infiltration of each sample by calculating the TILs proportion which refers to lymphocyte counts divided by the total cell counts.

### Statistical analysis

The distributions of the patient’s demographic characteristics were summarized and presented as counts and percentages. Differences in ordered categorical or continuous variables between cancer types were assessed with the Kruskal-Wallis H test and Wilcoxon rank sum tests. The Pearson chi-square test was used for disordered categorical variables. The multiple comparisons were corrected by the Bonferroni method. The correlations between the auto-assessed TILs percentage and demographic information were analyzed using a Kruskal-Wallis H test since the proportion of TILs did not meet the prerequisites for the parametric testing method. Spearman correlation analysis and multi-variable linear regression were performed to explore the distribution of TILs proportion between cancer types with adjustment for age, sex, body mass index (BMI), first-line treatment method, tumor differentiation grade, tumor-node-metastasis (TNM) staging, i.e., factors correlated with TILs levels. Besides, we also adjusted for *Helicobacter pylori* infection status, pickles intake, and tea drinking which were significant in univariable correlation analyses.

Overall survival (OS) was defined as the time from diagnosis to the time of any-cause death or the last follow-up. The OS was evaluated with Kaplan-Meier curves and compared using log-rank tests. The Cox proportional hazard model was conducted for univariable and multivariable association analyses between the auto-quantified TILs proportion and OS, adjusted for age, sex, BMI, first-line treatment method, and tumor differentiation grade, i.e., factors associated with esophagogastric cancers survival. The multivariable model adjusting for TNM staging and other demographic characteristics was used for the 117 AEGJ, 148 GAC, and 418 ESCC cases with TNM staging information. The hazard ratio (HR) and 95% confidence interval (CI) were estimated. The statistical analyses were performed using R version 4.1.2 (http://cran.r-project.org). Two-sided *P* < 0.05 was considered statistically significant.

### Availability of data and materials

The datasets generated during and analyzed during the current study are available from the corresponding author upon reasonable request.

## Supplementary Materials

Supplementary Figures

Supplementary Tables

Supplementary Dataset 1

Supplementary Dataset 2

Supplementary Dataset 3
