# Supplementary material for: Characteristics of auto-quantified tumor-infiltrating lymphocytes and the prognostic value in adenocarcinoma of the esophagogastric junction, gastric adenocarcinoma, and esophageal squamous cell carcinoma
Source: Aging (Albany NY). 2024 Jul 5;16(13):11027–61. doi: 10.18632/aging.205999 (PMC11272125; doi:10.18632/aging.205999)
Supplement: Supplementary Figures [file aging-16-205999-s001.pdf]

SUPPLEMENTARY FIGURES

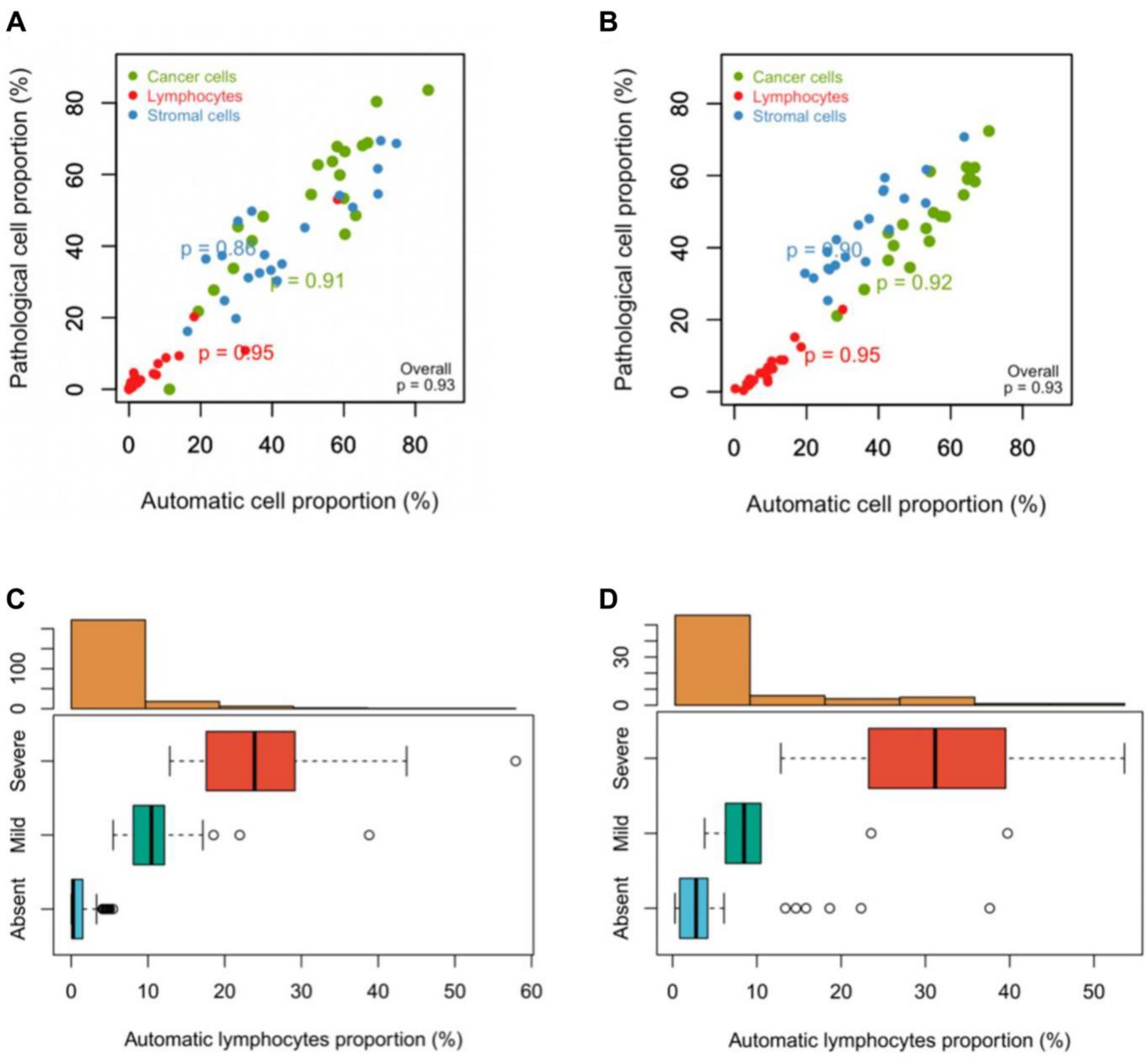

Supplementary Figure 1. Verifications of the cell training sets. (**A**, **B**) Cell proportions obtained by automated image analysis were compared to a pathologist's counts for a total of 10,000 single cells in the two representative sets of 20 ESCC and 20 GAC tissue samples. (**C**, **D**) TIL proportions versus manual grading of TIL infiltration in random one-third samples of ESCC and GAC.

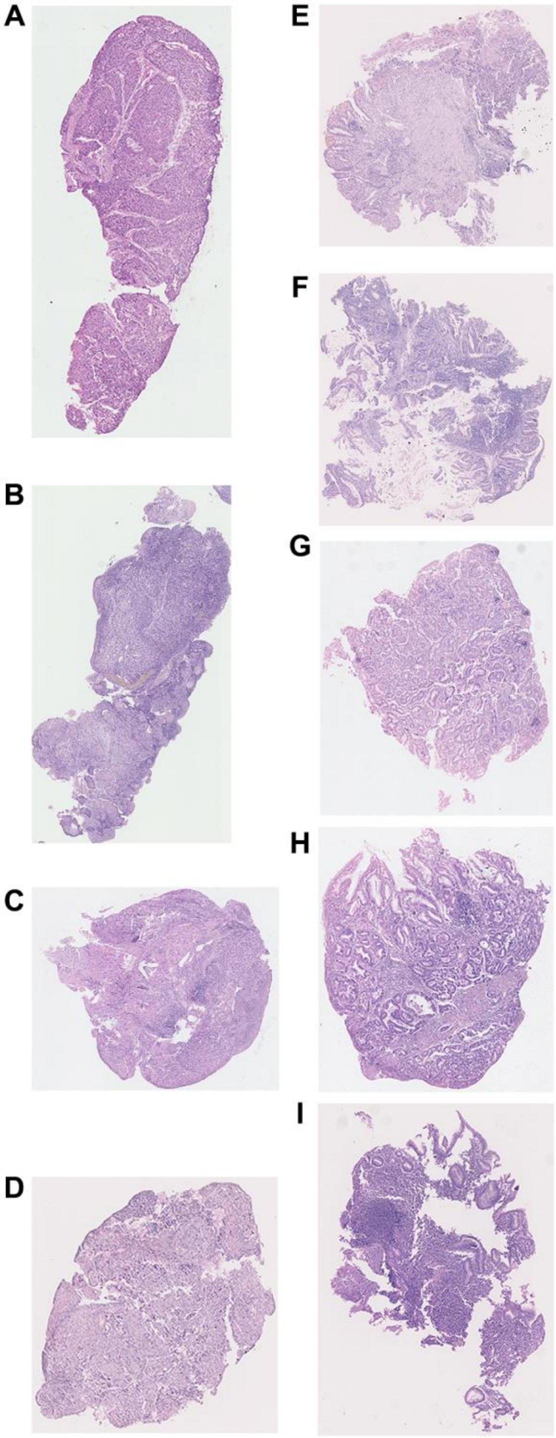

Supplementary Figure 2. The raw images of TILs intensity examples in ESCC, AEGJ, and GAC H&E-stained tissue sections (H&E×200). (**A**–**C**) The H&E-stained tumor tissue sections with low TILs grade (0.00%, 0.02%), medium TILs grade (0.02%, 0.75%), and high TILs grade (0.75%, 57.92%) infiltration in ESCC. (**D**–**F**) The H&E-stained tumor tissue sections with low TILs grade (0.01%, 1.87%), medium TILs grade (1.87%, 11.14%), and high TILs grade (11.14%, 53.89%) infiltration in AEGJ. (**G**–**I**) The H&E-stained tumor tissue sections with low TILs grade (0.00%-0.43%), medium TILs grade (0.43%, 8.54%), and high TILs grade (8.54%, 75.05%) infiltration in GAC.

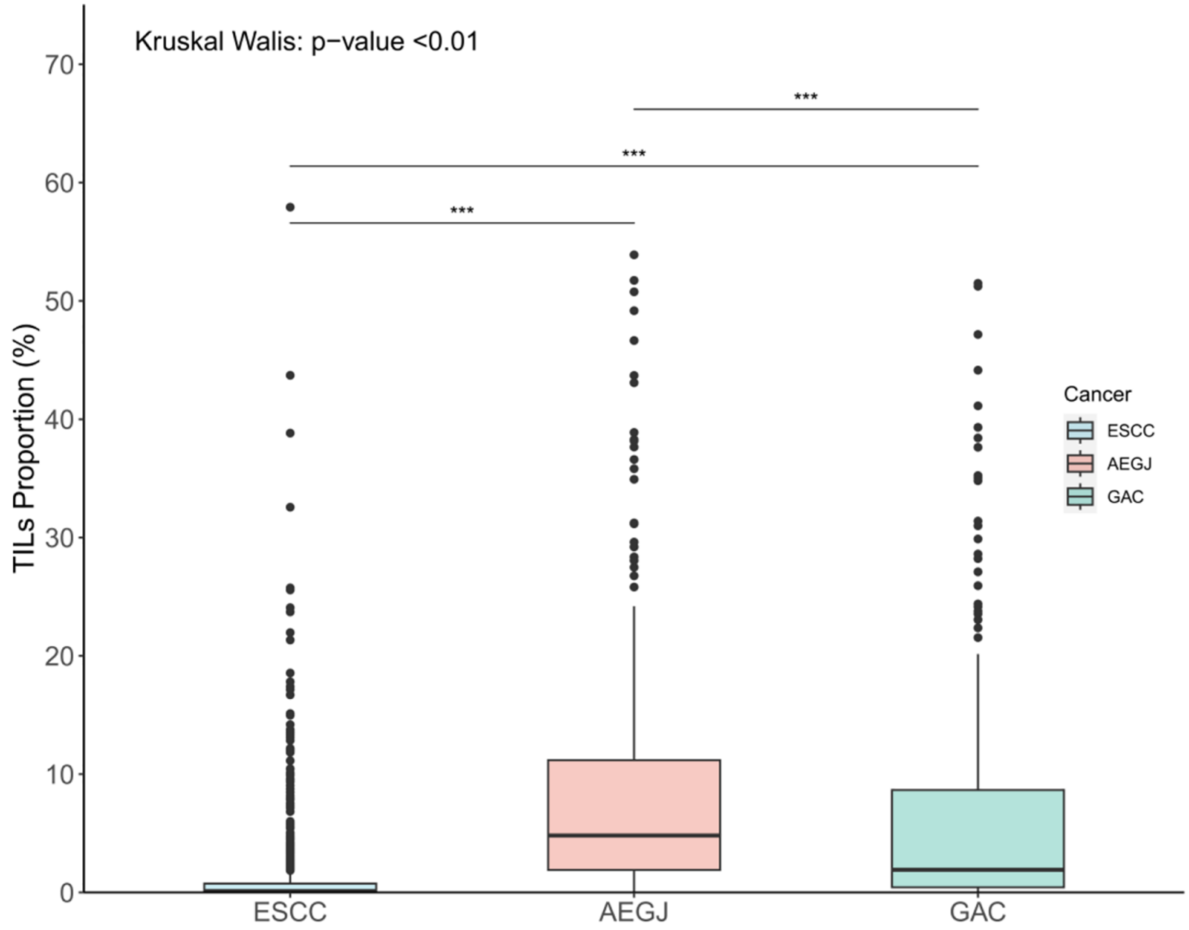

Supplementary Figure 3. Comparison of the TILs proportions in 752 ESCC, 214 AEGJ, and 256 GAC cases without stratification.
