# Supplementary material for: Characteristics of auto-quantified tumor-infiltrating lymphocytes and the prognostic value in adenocarcinoma of the esophagogastric junction, gastric adenocarcinoma, and esophageal squamous cell carcinoma
Source: Aging (Albany NY). 2024 Jul 5;16(13):11027–61. doi: 10.18632/aging.205999 (PMC11272125; doi:10.18632/aging.205999)
Supplement: Supplementary Tables [file aging-16-205999-s002.pdf]

SUPPLEMENTARY TABLES

Supplementary Table 1. Classification accuracy by 2-fold cross-validation for cancer cells, lymphocytes, and stromal cells based on the AEGJ, GAC, and ESCC training and testing sets.

Classes	SVM Prediction (Training set)				SVM Prediction (Testing set)			
	Cancer	Lymphocyte	Stromal	Recall (%)	Cancer	Lymphocyte	Stromal	Recall (%)
**AEGJ**								
Cancer	426	28	34	87.30	463	14	35	90.43
Lymphocyte	23	425	7	93.41	18	408	3	95.78
Stromal	58	4	187	75.10	38	4	209	83.27
Precision (%)	84.02	93.00	82.02		89.21	95.77	84.62	
**GAC**								
Cancer	530	1	4	99.07	523	3	5	98.40
Lymphocyte	9	748	1	98.68	4	729	0	99.45
Stromal	12	0	189	94.03	10	0	220	95.65
Precision (%)	96.19	99.87	97.42		97.39	99.59	97.78	
**ESCC**								
Cancer	410	4	98	80.08	438	4	91	82.18
Lymphocyte	3	166	48	76.50	4	167	34	81.46
Stromal	11	7	594	97.06	19	15	569	94.36
Precision (%)	96.70	93.79	80.27		95.01	89.78	81.99	

The confusion matrix of the real classes of the cells (columns) and the predicted classes (rows) are shown, where Precision was calculated by true positives/(true positives + false positives) and Recall was calculated by true positives/(true positives + false negatives).

Supplementary Table 2. The comparison of TILs proportion in the 214 AEGJ, 256 GAC, and 752 ESCC cases.

Variables	TILs proportion, Median (IQR)			*P*-value
	AEGJ (%)	GAC (%)	ESCC (%)	
**All patients**	4.82 (1.90, 11.18)	1.92 (0.44, 8.66)	0.12 (0.02, 0.75)	**<0.001**
**Age**				
<60	3.82 (1.57, 11.05)	1.86 (0.16, 9.16)	0.15 (0.03, 0.92)	**<0.001**
≥60	4.88 (2.01, 11.21)	1.93 (0.47, 8.16)	0.12 (0.02, 0.73)	**<0.001**
**Sex**				
Man	4.55 (1.71, 10.80)	2.38 (0.52, 9.14)	0.12 (0.02, 0.67)	**<0.001**
Woman	5.19 (2.41, 12.45)	1.27 (0.30, 6.54)	0.13 (0.02, 1.10)	**<0.001**
**BMI**				
<18.5	5.02 (1.32, 8.99)	1.83 (0.33, 6.88)	0.08 (0.02, 0.56)	**<0.001**
18.5-24	4.56 (1.99, 11.21)	2.34 (0.51, 8.08)	0.11 (0.02, 0.62)	**<0.001**
≥24	4.97 (2.09, 10.82)	1.86 (0.43, 9.15)	0.23 (0.02, 1.20)	**<0.001**
**Tea drinking**				
Never	4.65 (1.56, 11.01)	1.51 (0.29, 7.93)	0.12 (0.02, 0.72)	**<0.001**
Ever	5.36 (3.02, 11.11)	2.74 (1.14, 10.47)	0.12 (0.02, 0.75)	**<0.001**
**Times of tooth brushing daily**				
<2	4.94 (2.03, 11.21)	2.18 (0.46, 9.09)	0.12 (0.02, 0.75)	**<0.001**
≥2	3.98 (1.29, 6.62)	1.79 (0.33, 8.08)	0.12 (0.02, 0.75)	**<0.001**

**Wealth scores**				
Q1	4.99 (2.47, 11.56)	2.13 (0.37, 8.91)	0.12 (0.02, 0.69)	**<0.001**
Q2	2.94 (1.36, 5.60)	1.27 (0.36, 4.56)	0.13 (0.02, 0.96)	**<0.001**
Q3	5.22 (2.24, 13.93)	2.55 (0.38, 2.55)	0.09 (0.01, 0.45)	**<0.001**
Q4	4.26 (1.23, 10.69)	2.38 (0.67, 2.38)	0.13 (0.02, 0.51)	**<0.001**
Q5	5.71 (3.41, 13.55)	2.41 (0.93, 4.74)	0.25 (0.03, 2.05)	**<0.001**
Helicobacter pylori				
HP+	4.62 (1.84, 11.24)	2.28 (0.45, 9.66)	0.13 (0.02, 0.77)	**<0.001**
HP−	5.24 (2.99, 11.10)	1.83 (0.39, 6.87)	0.12 (0.02, 0.60)	**<0.001**
**Gastric atrophy**				
Yes	4.62 (1.84, 11.24)	2.28 (0.45, 9.66)	0.13 (0.02, 0.77)	**<0.001**
No	5.24 (2.99, 11.10)	1.83 (0.39, 6.87)	0.12 (0.02, 0.60)	**<0.001**
**First-line treatment method**				
Radiotherapy	2.77 (0.40, 7.72)	0.82 (0.27, 2.74)	0.09 (0.02, 0.68)	**<0.001**
Chemotherapy	4.10 (1.56, 5.30)	1.32 (0.30, 13.71)	0.10 (0.02, 0.48)	**<0.001**
Surgery	5.72 (3.14, 11.94)	2.78 (0.73, 11.12)	0.15 (0.03, 0.80)	**<0.001**
Combination therapy	4.64 (1.81, 10.42)	1.32 (0.35, 3.21)	0.10 (0.02, 0.92)	**<0.001**
Untreated	1.83 (1.05, 3.49)	0.66 (0.18, 1.61)	0.03 (0.02, 0.43)	**0.002**
**TNM staging**				
0+I+II	6.92 (3.12, 13.39)	2.64 (0.49, 10.91)	0.14 (0.02, 0.76)	**<0.001**
III+IV	5.34 (2.99, 11.56)	2.47 (0.74, 11.12)	0.15 (0.02, 0.92)	**<0.001**
**Grade of differentiation**				
Gx Grading cannot be evaluated	4.04 (1.77, 12.42)	2.38 (0.82, 10.20)	0.16 (0.02, 1.30)	**<0.001**
G1 Highly differentiated	8.67 (2.55, 14.28)	1.60 (0.68, 10.19)	0.13 (0.03, 0.69)	**0.008**
G2 Medium differentiation	7.72 (3.89, 13.39)	2.78 (1.13, 9.15)	0.13 (0.02, 0.84)	**<0.001**
G3 Poorly differentiated	7.17 (1.97, 12.81)	2.10 (0.26, 11.47)	0.11 (0.02, 0.56)	**<0.001**
G4 Undifferentiated	4.34 (2.42, 5.17)	0.85 (0.35, 3.50)	0.13 (0.05, 0.35)	**<0.001**

Supplementary Table 3. Univariable and multivariable Cox regression analyses of basic characteristics with OS in GAC of Taixing dataset (Discovery, *N* = 256).

Characteristics	No. of patients (%)	Median survival (years)	Univariable analysis		Multivariable analysis	
			P-value	HR (95% CI)	*P*-value	aHR[a] (95% CI)
**TILs, Median (IQR)**	1.92 (0.44, 8.66)		**0.030**	0.979 (0.961, 0.998)	**0.016**	0.972 (0.949, 0.995)
**Age, mean (range)**	67.52 (41–85)		**0.023**	1.022 (1.003, 1.041)	**0.919**	1.001 (0.977, 1.026)
**Sex**						
Woman	67 (26.17)	2.16		Ref.		Ref.
Man	189 (73.83)	2.65	0.061	0.711 (0.500, 1.016)	0.574	0.854 (0.493, 1.479)
**Marriage**						
Divorce/widow	61 (23.83)	2.56		Ref.		Ref.
Unmarried	9 (3.52)	0.98	0.103	1.955 (0.874, 4.375)	0.390	1.790 (0.475, 6.746)
Married	186 (72.66)	2.71	0.108	0.740 (0.512, 1.068)	0.665	1.135 (0.640, 2.011)
**Educational level**						
High school and above	18 (7.03)	3.47		Ref.		Ref.
Primary or Secondary school	158 (61.72)	2.64	0.095	2.038 (0.883, 4.705)	**0.027**	3.871 (1.163, 12.881)
Illiteracy	80 (31.25)	2.16	**0.027**	2.602 (1.113, 6.079)	0.077	3.272 (0.881, 12.148)

**Cigarette smoking**						
Never	103 (40.23)	2.16		Ref.		Ref.
Ever or still	144 (56.25)	2.73	0.077	0.737 (0.525, 1.034)	0.497	0.776 (0.373, 1.614)
Missing	9 (3.52)					
**Alcohol drinking**						
Never	125 (48.83)	2.64		Ref.		Ref.
Ever or still	122 (47.66)	2.62	0.536	0.899 (0.642, 1.259)	0.552	1.189 (0.673, 2.101)
Missing	9 (3.52)					
**Tea drinking**						
Never	170 (66.41)	2.64		Ref.		Ref.
Ever	77 (30.08)	2.48	0.856	0.967 (0.672, 1.391)	0.070	1.671 (0.960, 2.909)
Missing	9 (3.52)					
**BMI**						
≥24	66 (25.78)	3.01		Ref.		Ref.
18.5–24	158 (61.72)	2.55	0.434	1.176 (0.783, 1.766)	0.489	1.230 (0.684, 2.211)
<18.5	32 (12.50)	2.25	0.107	1.564 (0.908, 2.695)	0.103	1.938 (0.875, 4.294)
Missing	0 (0.00)					
**Wealth scores**						
Q5	40 (15.63)	2.64		Ref.		Ref.
Q4	47 (18.36)	2.68	0.448	0.792 (0.434, 1.447)	0.903	1.056 (0.438, 2.548)
Q3	62 (24.42)	3.14	0.453	0.810 (0.468, 1.403)	0.850	1.081 (0.484, 2.415)
Q2	55 (21.48)	1.99	0.508	1.202 (0.697, 2.070)	0.532	1.296 (0.574, 2.926)
Q1	52 (20.31)	2.40	0.321	1.309 (0.769, 2.227)	0.990	1.005 (0.461, 2.188)
**First-line treatment method**						
Untreated	11 (4.30)	0.81		Ref.		Ref.
Combination therapy	57 (22.27)	2.32	**0.003**	0.344 (0.169, 0.698)	**0.007**	0.175 (0.049, 0.620)
Surgery	151 (58.98)	3.00	**<0.001**	0.193 (0.098, 0.380)	**<0.001**	0.140 (0.043, 0.451)
Chemotherapy	27 (10.55)	0.86	0.658	0.844 (0.399, 1.786)	0.241	0.472 (0.135, 1.656)
Radiotherapy	9 (3.52)	1.21	0.413	0.681 (0.271, 1.709)	0.778	0.817 (0.202, 3.314)
Missing	1 (0.39)					
**Grade of differentiation**						
G1 Highly differentiated	7 (2.73)	3.59		Ref.		Ref.
G2 Medium differentiation	63 (24.61)	3.14	0.762	0.795 (0.181, 3.503)	0.799	0.822 (0.183, 3.694)
G3 Poorly differentiated	48 (18.75)	2.40	0.162	2.786 (0.663, 11.711)	0.162	2.835 (0.657, 12.237)
G4 Undifferentiated	9 (3.52)	3.48	0.320	2.300 (0.446, 11.868)	0.435	1.979 (0.357, 10.977)
Gx Grading cannot be evaluated	47 (18.36)	1.99	0.071	3.733 (0.893, 15.603)	0.242	2.467 (0.543, 11.209)
Missing	82 (32.03)					
Helicobacter pylori						
HP−	68 (26.56)	2.27		Ref.		Ref.
HP+	181 (70.70)	2.65	0.138	0.761 (0.531, 1.092)	0.543	0.836 (0.470, 1.488)
Missing	7 (2.73)					
**Gastric atrophy**						
No	157 (61.33)	2.71		Ref.		Ref.
Yes	51 (19.92)	2.10	**0.017**	1.623(1.091, 2.414)	**0.006**	2.183 (1.246, 3.826)
Missing	48 (18.75)					

[a]aHR with adjustment for TILs, age, sex, grade of differentiation, first-line treatment method, BMI.

Supplementary Table 4. Univariable and multivariable Cox regression analyses of basic characteristics with OS in ESCC of Taixing dataset (Discovery, *N* = 752).

Characteristics	No. of patients (%)	Median survival (years)	Univariable analysis		Multivariable analysis	
			P-value	HR (95% CI)	*P*-value	aHR[a] (95% CI)
**TILs, Median (IQR)**	0.12 (0.02, 0.75)		**0.008**	0.960 (0.931, 0.990)	**0.032**	0.967 (0.938, 0.997)
**Age, mean (range)**	66.96 (42–85)		**<0.001**	1.033 (1.021, 1.045)	**<0.001**	1.022 (1.010, 1.035)
**Sex**						
Woman	260 (34.57)	2.62		Ref.		Ref.
Man	492 (65.43)	2.35	**0.107**	1.180 (0.965, 1.444)	**0.020**	1.289 (1.040, 1.599)
**Marriage**						
Divorce/widow	136 (18.09)	2.54		Ref.		Ref.
Unmarried	25 (3.32)	1.31	**0.003**	2.105 (1.286, 3.445)	**0.037**	1.798 (1.036, 3.121)
Married	591 (78.59)	2.46	0.938	0.990 (0.773, 1.269)	0.346	1.137 (0.870, 1.486)
**Educational level**						
High school and above	47 (6.25)	2.43		Ref.		Ref.
Primary or Secondary school	429 (57.05)	2.61	0.796	1.057 (0.694, 1.609)	0.880	1.034 (0.669, 1.598)
Illiteracy	276 (36.70)	2.06	0.135	1.385 (0.903, 2.122)	0.190	1.374 (0.854, 2.210)
**Cigarette smoking**						
Never	313 (41.62)	2.59		Ref.		Ref.
Ever or still	405 (53.86)	2.40	0.600	1.053 (0.867, 1.280)	0.423	0.884 (0.653, 1.120)
Missing	34 (4.52)					
**Alcohol drinking**						
Never	351 (46.68)	2.43		Ref.		Ref.
Ever or still	366 (48.67)	2.45	0.523	0.939 (0.775, 1.139)	0.208	0.849 (0.658, 1.096)
Missing	35 (4.65)					
**Tea drinking**						
Never	493 (65.56)	2.51		Ref.		Ref.
Ever	225 (29.92)	2.33	0.483	1.076 (0.876, 1.322)	0.181	1.180 (0.926, 1.504)
Missing	34 (4.52)					
**BMI**						
≥24	201 (26.73)	2.77		Ref.		Ref.
18.5-24	466 (61.97)	2.22	**0.007**	1.377 (1.093, 1.735)	0.058	1.259 (0.993, 1.598)
<18.5	85 (11.30)	1.75	**0.001**	1.771 (1.281, 2.449)	0.063	1.378 (0.983, 1.932)
Missing	0 (0.00)					
**Wealth scores**						
Q5	62 (8.24)	2.67		Ref.		Ref.
Q4	138 (18.35)	2.63	0.232	1.262 (0.862, 1.848)	0.287	1.235 (0.837, 1.822)
Q3	161 (21.41)	2.49	0.240	1.253 (0.860, 1.826)	0.580	1.114 (0.760, 1.635)
Q2	139 (18.48)	2.31	0.158	1.318 (0.898, 1.935)	0.112	1.373 (0.928, 2.029)
Q1	232 (30.85)	2.18	**0.030**	1.480 (1.039, 2.109)	0.254	1.235 (0.859, 1.776)
**First-line treatment method**						
Untreated	21 (2.79)	0.89		Ref.		Ref.
Radiotherapy	88 (11.70)	1.68	**<0.001**	0.328 (0.199, 0.540)	**0.001**	0.391 (0.224, 0.683)
Chemotherapy	93 (12.37)	1.49	**<0.001**	0.368 (0.225, 0.602)	**0.004**	0.448 (0.259, 0.775)
Surgery	377 (50.13)	2.88	**<0.001**	0.166 (0.105, 0.263)	**<0.001**	0.236 (0.140, 0.399)
Combination therapy	170 (22.61)	1.84	**<0.001**	0.291 (0.182, 0.466)	**0.001**	0.400 (0.236, 0.678)
Missing	3 (0.40)					

Characteristics	No. of patients (%)	Median survival (years)	P-value	HR (95% CI)	P-value	aHR[a] (95% CI)
**Grade of differentiation**						
G1 Highly differentiated	60 (7.98)	2.85		Ref.		Ref.
G2 Medium differentiation	452 (60.11)	2.61	0.091	1.426 (0.945, 2.152)	0.142	1.366 (0.9005, 2.073)
G3 Poorly differentiated	134 (17.82)	2.05	**0.003**	1.966 (1.260, 3.069)	0.101	1.463 (0.929, 2.303)
G4 Undifferentiated	45 (5.98)	1.51	**0.003**	2.215 (1.302, 3.770)	0.087	1.607 (0.933, 2.768)
Gx Grading cannot be evaluated	40 (5.32)	1.68	**0.003**	2.250 (1.317, 3.845)	**0.031**	1.814 (1.055, 3.117)
Missing	21 (2.79)					
Helicobacter pylori						
HP−	234 (31.12)	2.45		Ref.		Ref.
HP+	484 (64.36)	2.38	0.271	1.124 (0.913, 1.383)	0.355	1.108 (0.892, 1.376)
Missing	34 (4.52)					
**Gastric atrophy**						
No	520 (69.15)	2.47		Ref.		Ref.
Yes	98 (13.03)	2.31	0.635	1.070 (0.808, 1.418)	0.468	1.114 (0.832, 1.490)
Missing	134 (17.82)					

[a]aHR with adjustment for TILs, age, sex, grade of differentiation, first-line treatment method, BMI.

Supplementary Table 5. Univariable and multivariable Cox regression analyses of basic characteristics with OS in AEGJ of Taixing dataset (Discovery, *N* = 117).

Characteristics	No. of patients (%)	Median survival (years)	Univariable analysis		Multivariable analysis	
			P-value	HR (95% CI)	*P*-value	aHR[a] (95% CI)
**TILs, Median (IQR)**	5.42 (3.04, 12.86)		**0.009**	0.957 (0.926, 0.989)	**0.009**	0.946 (0.907, 0.986)
**Age, mean (range)**	69 (49–84)		0.546	1.010 (0.978, 1.044)	0.553	1.012 (0.973, 1.053)
**Sex**						
Woman	35 (29.91)	2.56		Ref.		Ref.
Man	82 (70.09)	2.82	0.628	0.874 (0.507, 1.506)	0.605	0.849 (0.458, 1.577)
**Marriage**						
Divorce/widow	28 (23.93)	3.05		Ref.		Ref.
Unmarried	9 (7.69)	2.56	0.579	0.703 (0.202, 2.448)	0.073	0.149 (0.019, 1.194)
Married	80 (68.38)	2.71	0.600	1.175 (0.643, 2.147)	0.712	0.857 (0.379, 1.938)
**Educational level**						
High school and above	6 (5.13)	2.59		Ref.		Ref.
Primary or Secondary school	66 (56.41)	2.83	0.285	0.597 (0.232, 1.537)	0.407	0.637 (0.219, 1.851)
Illiteracy	45 (38.46)	2.70	0.520	0.729 (0.278, 1.909)	0.608	0.720 (0.205, 2.524)
**Cigarette smoking**						
Never	45 (38.46)	3.26		Ref.		Ref.
Ever or still	70 (59.83)	2.53	0.331	1.298 (0.767, 2.198)	0.322	1.364 (0.738, 2.522)
Missing	2 (1.71)					
**Alcohol drinking**						
Never	70 (59.83)	3.11		Ref.		Ref.
Ever or still	45 (38.46)	2.53	0.790	1.073 (0.637, 1.809)	0.755	0.912 (0.511, 1.628)
Missing	2 (1.71)					
**Tea drinking**						
Never	88 (75.21)	2.84		Ref.		Ref.
Ever	27 (23.08)	2.17	0.480	1.235 (0.688, 2.218)	0.822	1.079 (0.555, 2.101)
Missing	2 (1.71)					

**BMI**						
≥24	27 (23.08)	2.93		Ref.		Ref.
18.5-24	78 (66.67)	2.75	0.792	0.925 (0.519, 1.649)	0.434	0.841 (0.423, 1.674)
<18.5	11 (9.40)	2.70	0.493	0.681 (0.228, 2.040)	0.435	0.625 (0.193, 2.030)
Missing	1 (0.85)					
**Wealth scores**						
Q5	14 (11.97)	2.98		Ref.		Ref.
Q4	24 (20.51)	2.25	0.391	1.521 (0.584, 3.963)	0.636	0.769 (0.260, 2.279)
Q3	24 (20.51)	2.60	0.572	1.322 (0.502, 3.481)	0.959	0.972 (0.327, 2.894)
Q2	22 (18.80)	3.67	0.621	1.277 (0.485, 3.365)	0.893	0.931 (0.328, 2.642)
Q1	33 (28.21)	2.88	0.987	1.008 (0.391, 2.600)	0.661	0.792 (0.279, 2.247)
**First-line treatment method**						
Untreated	1 (0.85)	1.58		Ref.		Ref.
Radiotherapy	4 (3.42)	1.16	0.744	0.684 (0.070, 6.676)	0.969	0.946 (0.059, 15.110)
Chemotherapy	4 (3.42)	2.08	0.859	0.819 (0.091, 7.400)	0.990	1.015 (0.108, 9.568)
Surgery	82 (70.09)	2.82	0.226	0.291 (0.039, 2.150)	0.900	1.153 (0.125, 10.65)
Combination therapy	26 (22.22)	3.03	0.102	0.176 (0.022, 1.415)	0.731	0.663 (0.064, 6.916)
Missing	0 (0.00)					
TNM staging						
0+I+II	55 (47.01)	2.94		Ref.		Ref.
III+IV	62 (52.99)	2.14	**0.027**	1.795 (1.068, 3.014)	0.200	1.462 (0.813, 2.611)
**Grade of differentiation**						
G1 Highly differentiated	4 (3.42)	2.19		Ref.		Ref.
G2 Medium differentiation	61 (52.14)	3.00	0.574	0.661 (0.156, 2.797)	0.771	0.804 (0.184, 3.512)
G3 Poorly differentiated	26 (22.22)	2.73	0.979	0.980 (0.223, 4.319)	0.919	1.082 (0.234, 4.998)
G4 Undifferentiated	8 (6.84)	0.68	0.087	3.971 (0.819, 19.250)	0.100	4.242 (0.757, 23.762)
Gx Grading cannot be evaluated	14 (11.97)	1.70	0.378	1.971 (0.436, 8.907)	0.365	2.128 (0.416, 10.891)
Missing	4 (3.42)					
Helicobacter pylori						
HP−	19 (16.24)	2.70		Ref.		Ref.
HP+	95 (81.20)	2.80	0.987	0.994 (0.503, 1.964)	0.968	0.983 (0.419, 2.251)
Missing	3 (2.56)					
**Gastric atrophy**						
No	70 (59.83)	2.66		Ref.		Ref.
Yes	29 (24.79)	2.76	0.461	1.234 (0.706, 2.159)	0.786	0.914 (0.475, 1.755)
Missing	18 (15.38)					

[a]aHR with adjustment for TILs, age, sex, grade of differentiation, TNM staging, first-line treatment method, BMI.

Supplementary Table 6. Univariable and multivariable Cox regression analyses of basic characteristics with OS in GAC of Taixing dataset (Discovery, *N* = 148).

Characteristics	No. of patients (%)	Median survival (years)	Univariable analysis		Multivariable analysis	
			P-value	HR (95% CI)	*P*-value	aHR[a] (95% CI)
**TILs, Median (IQR)**	2.55 (0.51, 11.06)		0.192	0.983 (0.958, 1.009)	**0.016**	0.961 (0.931, 0.993)
**Age, mean (range)**	65.22 (44–85)		0.853	1.003 (0.974, 1.032)	0.600	0.990 (0.955, 1.027)

**Sex**						
Woman	39 (26.35)	2.88		Ref.		Ref.
Man	109 (73.65)	2.75	0.982	0.993 (0.568, 1.736)	0.896	0.955 (0.483, 1.891)
**Marriage**						
Divorce/widow	31 (20.95)	2.88		Ref.		Ref.
Unmarried	3 (2.03)	2.01	0.364	1.987 (0.452, 8.740)	0.321	0.319 (0.034, 3.046)
Married	114 (77.03)	2.87	0.608	0.858 (0.478, 1.540)	0.766	1.131 (0.503, 2.546)
**Educational level**						
High school and above	14 (9.46)	3.42		Ref.		Ref.
Primary or Secondary school	95 (64.19)	2.81	0.262	1.962 (0.605, 6.362)	**0.045**	4.728 (1.032, 21.662)
Illiteracy	39 (26.35)	2.82	0.111	2.679 (0.798, 9.001)	**0.043**	5.534 (1.056, 28.992)
**Cigarette smoking**						
Never	59 (39.86)	2.87		Ref.		Ref.
Ever or still	88 (59.46)	2.82	0.746	0.920 (0.555, 1.524)	0.101	0.496 (0.214, 1.148)
Missing	1 (0.68)					
**Alcohol drinking**						
Never	75 (50.68)	2.98		Ref.		Ref.
Ever or still	72 (48.65)	2.64	0.492	1.191 (0.723, 1.961)	0.440	1.390 (0.602, 3.210)
Missing	1 (0.68)					
**Tea drinking**						
Never	100 (67.57)	2.91		Ref.		Ref.
Ever	47 (31.76)	2.46	0.139	1.477 (0.882, 2.473)	0.299	1.475 (0.708, 3.070)
Missing	1 (0.68)					
**BMI**						
≥24	43 (29.05)	3.14		Ref.		Ref.
18.5-24	90 (60.81)	2.64	0.513	1.215 (0.677, 2.181)	0.188	1.678 (0.777, 3.626)
<18.5	15 (10.14)	2.75	0.369	1.477 (0.631, 3.454)	0.135	2.193 (0.783, 6.143)
Missing	0 (0.00)					
**Wealth scores**						
Q5	22 (14.86)	2.62		Ref.		Ref.
Q4	30 (20.27)	2.97	0.479	0.733 (0.311, 1.731)	0.648	0.792 (0.291, 2.158)
Q3	40 (27.03)	3.34	0.174	0.564 (0.246, 1.289)	0.187	0.518 (0.195, 1.376)
Q2	30 (20.27)	2.44	0.744	0.869 (0.375, 2.015)	0.477	0.698 (0.259, 1.881)
Q1	26 (17.57)	2.41	0.527	1.291 (0.585, 2.848)	0.416	0.668 (0.252, 1.768)
**First-line treatment method**						
Untreated	2 (1.35)	1.19		Ref.		Ref.
Radiotherapy	3 (2.03)	0.98	0.997	1.004 (0.167, 6.039)	0.070	0.043 (0.001, 1.276)
Chemotherapy	11 (7.43)	0.93	0.942	0.945 (0.206, 4.342)	**0.031**	0.026 (0.001, 0.723)
Surgery	106 (7.16)	3.11	**0.004**	0.120 (0.028, 0.509)	**0.002**	0.007 (0.001, 0.161)
Combination therapy	25 (16.89)	2.64	**0.028**	0.180 (0.039, 0.828)	**0.005**	0.010 (0.001, 0.253)
Missing	1 (0.68)					
**TNM staging**						
0+I+II	98 (66.22)	3.27		Ref.		Ref.
III+IV	50 (33.78)	1.85	**<0.001**	4.568 (2.733, 7.633)	**0.001**	3.115 (1.546, 6.278)
**Grade of differentiation**						
G1 Highly differentiated	7 (4.73)	3.59		Ref.		Ref.
G2 Medium differentiation	60 (40.54)	3.14	0.755	0.789 (0.178, 3.501)	0.643	0.696 (0.151, 3.218)

G3 Poorly differentiated	40 (27.03)	2.55	0.225	2.460 (0.575, 10.519)	0.673	1.407 (0.288, 6.878)
G4 Undifferentiated	7 (4.73)	3.48	0.315	2.460 (0.575, 10.519)	0.438	2.045 (0.335, 12.482)
Gx Grading cannot be evaluated	23 (15.54)	1.79	0.093	3.576 (0.809, 15.801)	0.922	1.089 (0.199, 5.957)
Missing	11 (7.43)					
Helicobacter pylori						
HP−	34 (22.97)	2.52		Ref.		Ref.
HP+	108 (72.97)	2.87	0.763	0.914 (0.510, 1.639)	0.721	1.141 (0.554, 2.349)
Missing	6 (4.05)					
**Gastric atrophy**						
No	90 (60.81)	3.14		Ref.		Ref.
Yes	30 (20.27)	2.10	**0.002**	2.547 (1.407, 4.609)	**0.047**	2.196 (1.011, 4.771)
Missing	28 (18.92)					

[a]aHR with adjustment for TILs, age, sex, grade of differentiation, TNM staging, first-line treatment method, BMI.

Supplementary Table 7. Univariable and multivariable Cox regression analyses of basic characteristics with OS in ESCC of Taixing dataset (Discovery, *N* = 418).

Characteristics	No. of patients (%)	Median survival (years)	Univariable analysis		Multivariable analysis	
			P-value	HR (95% CI)	*P*-value	aHR[a] (95% CI)
**TILs, Median (IQR)**	0.14 (0.02, 0.84)		**0.045**	0.958 (0.918, 0.999)	**0.041**	0.954 (0.911, 0.998)
**Age, mean (range)**	65.60 (42–85)		**0.003**	1.024 (1.008, 1.041)	**0.016**	1.021 (1.004, 1.039)
**Sex**						
Woman	152 (36.36)	2.89		Ref.		Ref.
Man	266 (63.64)	2.54	**0.023**	0.716 (0.537, 0.954)	**0.004**	1.574 (1.157, 2.140)
**Marriage**						
Divorce/widow	70 (16.75)	2.58		Ref.		Ref.
Unmarried	9 (2.15)	0.99	**0.019**	2.649 (1.172, 5.986)	**0.005**	3.765 (1.504, 9.424)
Married	339 (81.10)	2.74	0.697	1.076 (0.745, 1.553)	0.147	1.341 (0.902, 1.993)
**Educational level**						
High school and above	25 (5.98)	2.81		Ref.		Ref.
Primary or Secondary school	252 (60.29)	2.71	0.583	1.188 (0.642, 2.200)	0.787	1.091 (0.579, 2.055)
Illiteracy	141 (33.73)	2.61	0.416	1.300 (0.690, 2.449)	0.340	1.398 (0.702, 2.786)
**Cigarette smoking**						
Never	180 (43.06)	2.76		Ref.		Ref.
Ever or still	219 (52.39)	2.61	0.223	1.187 (0.901, 1.564)	0.179	0.745 (0.485, 1.144)
Missing	19 (4.55)					
**Alcohol drinking**						
Never	194 (46.41)	2.61		Ref.		Ref.
Ever or still	205 (49.04)	2.76	0.914	1.015 (0.773, 1.333)	0.094	0.735 (0.513, 1.053)
Missing	199 (4.55)					
**Tea drinking**						
Never	285 (68.18)	2.70		Ref.		Ref.
Ever	115 (27.51)	2.55	0.268	1.178 (0.881, 1.576)	0.749	1.058 (0.749, 1.493)
Missing	18 (4.31)					
**BMI**						
≥24	122 (29.19)	2.82		Ref.		Ref.
18.5–24	248 (59.33)	2.61	0.095	1.308 (0.955, 1.791)	0.564	0.564 (0.796, 1.521)

<18.5	48 (11.48)	2.09	**0.047**	1.579 (1.006, 2.479)	0.406	0.406 (0.765, 1.937)
Missing	0 (0.00)					
**Wealth scores**						
Q5	46 (11.00)	2.92		Ref.		Ref.
Q4	84 (20.10)	2.66	0.149	1.489 (0.867, 2.558)	0.332	1.316 (0.755, 2.295)
Q3	78 (18.66)	2.57	0.429	1.258 (0.712, 2.223)	0.918	1.031 (0.575, 1.849)
Q2	85 (20.33)	2.62	0.259	1.373 (0.791, 2.382)	0.201	1.445 (0.822, 2.542)
Q1	125 (29.90)	2.68	0.068	1.615 (0.964, 2.706)	0.245	1.373 (0.804, 2.343)
**First-line treatment method**						
Untreated	2 (0.47)	0.38		Ref.		Ref.
Radiotherapy	39 (9.33)	2.25	**<0.001**	0.063 (0.014, 0.282)	**<0.001**	0.059 (0.128, 0.274)
Chemotherapy	46 (11.00)	1.53	**<0.001**	0.078 (0.018, 0.343)	**<0.001**	0.063 (0.139, 0.283)
Surgery	250 (59.81)	2.89	**<0.001**	0.040 (0.009, 0.171)	**<0.001**	0.054 (0.125, 0.236)
Combination therapy	81 (19.38)	2.55	**<0.001**	0.048 (0.011, 0.211)	**<0.001**	0.059 (1.331, 0.263)
Missing	0 (0.00)					
**TNM staging**						
0+I+II	256 (61.24)	3.04		Ref.		Ref.
III+IV	162(38.76)	1.56	**<0.001**	2.435 (1.863, 3.182)	**<0.001**	2.273 (1.684, 3.070)
**Grade of differentiation**						
G1 Highly differentiated	39 (9.33)	3.13		Ref.		Ref.
G2 Medium differentiation	283 (67.70)	2.76	0.067	1.701 (0.963, 3.003)	0.115	1.596 (0.893, 2.852)
G3 Poorly differentiated	51 (12.20)	1.75	**0.003**	2.611 (1.373, 4.966)	0.131	1.671 (0.859, 3.251)
G4 Undifferentiated	34 (8.13)	2.32	**0.009**	2.505 (1.253, 5.010)	0.589	1.223 (0.589, 2.542)
Gx Grading cannot be evaluated	9 (2.15)	1.59	**0.011**	3.286 (1.309, 8.247)	0.121	2.145 (0.818, 5.625)
Missing	2 (0.48)					
Helicobacter pylori						
HP−	135 (32.30)	2.61		Ref.		Ref.
HP+	263 (62.92)	2.66	0.328	1.156 (0.864, 1.547)	0.519	1.103 (0.818, 1.488)
Missing	20 (4.78)					
**Gastric atrophy**						
No	289 (69.14)	2.68		Ref.		Ref.
Yes	54 (12.92)	2.50	0.849	1.040 (0.695, 1.556)	0.750	1.071 (0.701, 1.638)
Missing	75 (17.94)					

[a]aHR with adjustment for TILs, age, sex, grade of differentiation, TNM staging, first-line treatment method, BMI.

Supplementary Table 8. Univariable and multivariable Cox regression analyses of basic characteristics with OS in AEGJ of TCGA dataset (Validation, *N* = 169).

Characteristics	No. of patients (%)	Median survival (years)	Univariable analysis		Multivariable analysis	
			P-value	HR (95% CI)	*P*-value	aHR[a] (95% CI)
**TILs, Median (IQR)**	1.99 (0.63, 6.38)		**0.003**	0.897 (0.835, 0.964)	**0.002**	0.812 (0.712, 0.925)
**Age, mean (range)**	65.43 (27–90)		0.806	1.003 (0.982, 1.024)	0.344	1.014 (0.985, 1.045)
**Sex**						
Woman	46 (27.22)	0.97		Ref.		Ref.
Man	123 (72.78)	1.36	0.530	1.207 (0.671, 2.169)	0.444	1.325 (0.646, 2.718)

**First-line treatment method**						
Pharmaceutical Therapy	89 (52.66)	1.25		Ref.		Ref.
Radiotherapy	80 (47.34)	1.26	0.613	0.887 (0.559, 1.409)	0.234	1.447 (0.788, 2.657)
**Grade of differentiation**						
Grade 1–2	46 (27.22)	1.45		Ref.		Ref.
Grade 3	72 (42.60)	1.08	0.073	1.787 (0.948, 3.366)	0.125	1.676 (0.866, 3.240)
Missing	51 (30.18)					
**TNM staging**						
I+II	71 (42.01)	1.28		Ref.		Ref.
III+IV	85 (50.30)	1.35	**0.014**	1.921 (1.140, 3.236)	0.644	1.167 (0.606, 2.249)
Missing	13 (7.69)					

[a]aHR with adjustment for TILs proportion, age, sex, first-line treatment method, grade of differentiation, and TNM staging.

Supplementary Table 9. Univariable and multivariable Cox regression analyses of basic characteristics with OS in GAC of TCGA dataset (Validation, *N* = 222).

Characteristics	No. of patients (%)	Median survival (years)	Univariable analysis		Multivariable analysis	
			P-value	HR (95% CI)	*P*-value	aHR[a] (95% CI)
**TILs, Median (IQR)**	4.14 (1.17, 12.99)		**0.004**	0.961 (0.935, 0.988)	**0.025**	0.969 (0.942, 0.996)
**Age, mean (range)**	65.52 (30–90)		**0.019**	1.027 (1.004, 1.050)	**0.002**	1.039 (1.014, 1.064)
**Sex**						
Woman	80 (27.22)	1.72		Ref.		Ref.
Man	142 (72.78)	1.17	0.492	1.172 (0.746, 1.841)	0.797	1.064 (0.662, 1.711)
**First-line treatment method**						
Pharmaceutical Therapy	109 (49.10)	1.30		Ref.		Ref.
Radiotherapy	113 (50.90)	1.44	0.873	0.966 (0.627, 1.486)	0.912	1.026 (0.646, 1.631)
**Grade of differentiation**						
Grade 1–2	83 (37.39)	1.44		Ref.		Ref.
Grade 3	133 (59.91)	1.18	0.085	1.510 (0.944, 2.414)	0.119	1.486 (0.904, 2.442)
Missing	6 (2.70)					
**TNM staging**						
I+II	95 (42.79)	1.66		Ref.		Ref.
III+IV	123 (55.41)	1.10	**0.001**	2.780 (1.704, 4.533)	**0.001**	2.464 (1.468, 4.134)
Missing	4 (1.80)					

[a]aHR with adjustment for TILs proportion, age, sex, first-line treatment method, grade of differentiation, and TNM staging.

Supplementary Table 10. Univariable and multivariable Cox regression analyses of basic characteristics with OS in ESCC of TCGA dataset (Validation, *N* = 70).

Characteristics	No. of patients (%)	Median survival (years)	Univariable analysis		Multivariable analysis	
			P-value	HR (95% CI)	*P*-value	aHR[a] (95% CI)
TILs, Median (IQR)	33.00 (9.77, 62.62)		0.080	0.986 (0.970, 1.002)	0.133	0.985 (0.965, 1.005)
Age, mean (range)	57.01 (36–90)		**0.031**	1.052 (1.005, 1.102)	0.058	1.063 (0.998, 1.132)
**Sex**						
Woman	11 (27.22)	1.95		Ref.		Ref.
Man	59 (72.78)	1.04	**0.045**	4.546 (1.032, 20.040)	0.070	6.690 (0.854, 52.415)

**First-line treatment method**						
Pharmaceutical Therapy	36 (70.00)	1.06		Ref.		Ref.
Radiotherapy	34 (50.90)	1.05	0.542	0.777 (0.346, 1.746)	0.187	0.492 (0.171, 1.411)
**Grade of differentiation**						
Grade 1-2	49 (70.00)	1.05		Ref.		Ref.
Grade 3	15 (21.43)	1.10	0.290	0.516 (0.152, 1.758)	0.182	0.357 (0.079, 1.619)
Missing	6 (8.57)					
**TNM staging**						
I+II	43 (61.43)	1.10		Ref.		Ref.
III+IV	26 (31.17)	1.02	**0.048**	2.258 (1.006, 5.067)	0.150	2.125 (0.762, 5.922)
Missing	1 (1.80)					

[a]aHR with adjustment for TILs proportion, age, sex, first-line treatment method, grade of differentiation, and TNM staging.
